# Urbanization Shifts Immunometabolism in a Common Bumblebee

**DOI:** 10.1002/ece3.70743

**Published:** 2024-12-22

**Authors:** Virginie Cuvillier‐Hot, Alessandro Fisogni, Vincent Doublet, Sylvain Guillot, Anne‐Catherine Holl, Julie Leclercq‐Dransart, Florent Occelli, Yves Piquot, Nina Hautekèete

**Affiliations:** ^1^ Univ Lille, CNRS, UMR 8198—Evo‐Eco‐Paleo Lille France; ^2^ Laboratory of Zoology Research Institute for Biosciences, University of Mons Mons Belgium; ^3^ Institute of Evolutionary Ecology and Conservation Genomics, University of Ulm Ulm Germany; ^4^ LGCgE, Laboratoire de Génie Civil et géo‐Environnement Univ. Lille, Univ. Artois, IMT Lille Douai, JUNIA, ULR 4515 Lille France

**Keywords:** *Bombus lapidarius*, eco‐immunology, gradient of urbanization, immune marker, urban stress, wild bees

## Abstract

The growing urbanization process is accompanied by the emergence of new habitats for wildlife, and cities are sometimes seen as refuges for pollinators such as wild bees compared to intensively cultivated rural habitats. However, the contrasting living conditions that combine high fragmentation, exposure to pollutants, and heat island effects, with low pesticide use and potentially high availability of resources, make it difficult to predict the overall effect of urban living on the health of wild bees. Moreover, if the responses of wild bee populations in terms of species richness and diversity have been the focus of many recent studies, individual responses to urbanization have been more rarely investigated. More specifically, data on the impacts on individual bee physiology and health are lacking. To help fill this gap, we collected red‐tailed bumblebee ( *Bombus lapidarius* ) workers along a gradient of urbanization defined by the level of soil imperviousness, and estimated environmental (air quality) and ecological (pathogens' prevalence and loads; local competition) pressures they locally experienced. In parallel, we quantified the expression of selected immune marker genes. We measured how the immune system of bumblebees responds to urbanization gradient and which local parameters best explain the observed changes in immune gene expression. We evidenced three immune markers, tightly linked with cellular metabolism, whose expressions increase with the level of urbanization, independently of individual infection and pollution exposure. We suggest that induction of their expression reveals a shift in wild bee immunometabolism, supposedly in response to the stressful conditions experienced in areas with high built‐up cover. The induction of these genes is likely at the root of any immune activation; they could thus be used as markers to estimate the levels of urban stress locally experienced by pollinators.

## Graphical abstract

Cities provide contrasting habitats for pollinators and it proves difficult to predict the overall effect of urban living on the health of wild bees. By combining measurements of local competition, pathogen pressures, and exposure to pollutants along an urbanization gradient, we evidenced an upregulation of genes involved in immunometabolism in bees living in areas of high built‐up cover, independently of the other local parameters measured. Such induction reflects a form of urban stress suffered locally by pollinators.

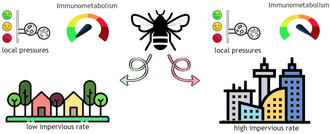

## 1 Introduction

Human footprint on a planetary scale is reflected in particular by galloping urbanization. The last IPBES assessment report on land degradation and restoration estimated that the current land occupied by built‐up areas increased by 17% between 2000 and 2010, and predicted a further 140% increase by 2030 (IPBES 2018). This rise in global urban land cover is fueled by rural‐to‐urban migration, especially in less developed countries, and by urban sprawling in wealthier regions. As human populations thrive, wildlife is on the cusp of a sixth mass extinction (IPBES 2019). A sharp decrease in insect biomass was concordantly reported by many studies (e.g., Biesmeijer et al. 2006; Cardoso et al. 2020; Hallmann et al. 2017, 2020; Thomas et al. 2004; Valtonen et al. 2017; van Klink et al. 2020; Wagner 2020; Wagner et al. 2021), and urbanization has been pinpointed as one of the negative drivers involved (e.g., Fenoglio, Rossetti, and Videla 2020; Piano et al. 2020). The effects of urban cover on wild insect populations are not easy to evaluate. Urban environments have their own biotic and abiotic characteristics, such as specific food resources or exposure to disturbance and diverse pollutions that affect the vital parameters of species in complex ways (Minias 2023; Rech et al. 2022), as well as specific ecological interactions (e.g., Rocha and Fellowes 2020; Theodorou 2022; Youngsteadt et al. 2015). The impacts of the multiple stressors of urban life on wildlife health are however poorly understood (Isaksson 2015), mainly because of the lack of health markers for each level of organization (community, population, individual).

In recent years, much attention has been paid to the decline of pollinators, with the importance of the ecosystem service of pollination now widely recognized (Murphy et al. 2022). The effects of urbanization on pollinator populations appear mixed in the recent literature. Several studies report positive influences, such as increased richness and diversity for some pollinator groups, especially in residential gardens, allotments, and higher income neighborhoods (Baldock et al. 2019, 2015; Theodorou et al. 2020); higher nest survival and density (Goulson et al. 2002); better health (Samuelson, Gill, and Leadbeater 2020); or higher reproductive success of focal species (Samuelson et al. 2018). In contrast, other studies highlight adverse effects on some species, related to imperviousness that limits ground nesting (Fauviau et al. 2022; Geslin et al. 2016; Herrmann, Buchholz, and Theodorou 2023; Milano et al. 2019; Persson et al. 2020), related to urban warming that can drive phenological mismatches with plant partners (Fisogni et al. 2020), and related to exposure to urban pollutants that negatively affect bee health (Thimmegowda et al. 2020). Such complexity of urban life effects on pollinators may come at least in part from the characteristics of the urban sites considered and in particular from their potential richness in green cover areas that can shelter bee‐friendly vegetation (Theodorou et al. 2020; Wenzel et al. 2020). Urban densification that leads to high percentages of built‐up surfaces, and virtually no space for high flower richness locally, would tend to drive population shrinkage. On the contrary, when it replaces landscapes of intensive agriculture, forming suburban areas, urban sprawl with a low degree of urbanization (below 50% impervious surface) can positively affect density and local pollinator richness (Wenzel et al. 2020). The criterion of urbanization level, most often approached by the percentage of impervious surfaces, therefore appears relevant for predicting the effect of the urban environment on local populations of pollinators (Fauviau et al. 2022; Herrmann, Buchholz, and Theodorou 2023) and is often used to investigate various population indicators (e.g., Fisogni et al. 2020, 2022; Fortel et al. 2014; Geslin et al. 2013). However, the effects of urbanization level at the individual scale have been more rarely addressed, and more specifically, data on the link with the physiology and health of wild pollinators are lacking.

Studies on bee health in an urban context have mainly focused on epidemiological aspects: urbanization tends to enhance the burden of pathogens in honeybees (Youngsteadt et al. 2015), as well as in different species of social bumblebees (BBs) (Goulson, Whitehorn, and Fowley 2012; Theodorou et al. 2016). Multiple environmental and ecological factors may elevate pathogen transmission and fitness in bee communities (Wilfert, Brown, and Doublet 2021). Increased promiscuity in urban areas, notably due to less numerous and more concentrated flower‐provisioning sites, may increase parasite transmission rate (amplification effect, Becker, Streicker, and Altizer 2015; Cohen et al. 2022). Flowers are recognized as parasite transmission hubs for pollinators (Durrer and Schmid‐Hempel 1994; Graystock et al. 2020; Graystock, Goulson, and Hughes 2015), and transmission is easier in species that form colonies of closely related individuals. However, higher parasite prevalence in highly urbanized areas may also result from impaired immunocompetence, both at the colony and individual levels, induced by the highly stressful urban environment. The present study aims to test this hypothesis by investigating the effect of urban stressors on immune activation in wild BBs.

BBs present several layers of defense against pathogens and parasites. The first layer is provided by their social lifestyle that offers behavioral defense mechanisms known as social immunity (Cremer, Armitage, and Schmid‐Hempel 2007), and is characterized by self‐medication, that is, the use of natural antimicrobial products present in plants, removal of diseased brood, or self‐isolation. The second layer of defense is the immune response expressed once a pathogen or parasite successfully penetrates the host. Although invertebrates lack an adaptive immune system, they present nevertheless an efficient innate immune system constituted of several conserved gene pathways expressed in response to diverse pathogenic agents (Hoffmann 1995). In honeybees, as demonstrated from a meta‐analysis of transcriptomes from *Apis mellifera* (Doublet et al. 2017), the transcriptional response to pathogens is composed of a core set of genes consistently activated against all types of pathogens, as well as more specific genes expressed against fungal or viral pathogens. This core set of genes may represent reliable markers of activation of the immune system and, by extension, of individual health status. In other words, individuals who exhibit significant modulations in the expression of these genes, in particular those upstream of the regulatory cascades, are more likely to have their immune system activated, thereby helping to fight infection and, potentially, leading to pathogen clearance. Such individuals will also bear associated costs (i.e., invested resources, oxidative damages), which likely explains why immune functions are so tightly regulated (Ashley, Weil, and Nelson 2012).

With this study, we aimed to test this scenario in urban wild pollinators, through a candidate gene approach, based on the knowledge acquired from honeybee transcriptomes. We collected BBs along an urbanization gradient defined by the increase in the percentage of impervious surfaces in a buffer of 500 m around flower‐rich sites, for which extensive insect and plant surveys were carried out in the same period (Fisogni et al. 2020, 2022). Local pressures experienced by BB populations have been assessed in various ways at each collection site: air quality was assessed following a lichen survey (species richness and thalli assays), whereas the intensity of local ecological interactions was estimated by a bee survey (abundances of *Bombus lapidarius* and *Apis mellifera* ) and by measuring parasite pressure (prevalence and loads of common BB pathogens/parasites). We focused on the red‐tailed BB ( *B. lapidarius* ), for its value in terms of ecosystem services and its potential as a good environmental indicator (Chowdhury et al. 2023). Indeed, the species is common in central European cities and is present in sufficient density even in city centers while remaining less successful than some other BB species, suggesting a certain sensitivity to urban deleterious effects. It also has a fairly limited foraging range around its nest (450 m, Knight et al. 2005), thus presenting itself as a good local sentinel species. Furthermore, no microevolutionary changes in response to urbanization were evidenced in the immune repertoire of this species (Theodorou et al. 2018), allowing to focus on the potential effects of urban life on the proximate immune mechanisms. Our questions were as follows: How is the immune system of collected BB affected along the urbanization gradient? Which local parameters among parasite pressure, pollution level, and level of soil imperviousness best explain the observed changes in immune gene expression?

## 2 Materials and Methods

### 2.1 Site Selection and Characterization

#### 2.1.1 Geographical Survey

This study was performed in the Metropole Européenne de Lille (MEL), in Northern France, characterized by a long history of strong anthropogenic pressures (Hautekèete et al. 2015). With more than 1 million inhabitants, the MEL is the fourth French agglomeration in terms of population. Although the MEL is deeply marked by its industrial past, agricultural surfaces remain the most represented in this area (Barles and Dumont 2021). Long‐term collaboration between scientists and the MEL Parks and Gardens Services allowed the identification of suitable urban meadows managed in the same way, in order to promote biodiversity, for at least 2 years prior to this study. These meadows were sown between 2010 and 2015 with standardized seed mixes containing only perennial herbaceous plants native to the region (purchased from Ecosem, Corroyle‐Grand, Belgium; see Fisogni et al. 2022, table A1, for the detailed list of plant species). For this study, we selected nine sites for which extensive insect and plant surveys were carried out in the same period (list of species in Fisogni et al. 2020, 2022).

**FIGURE 1:**
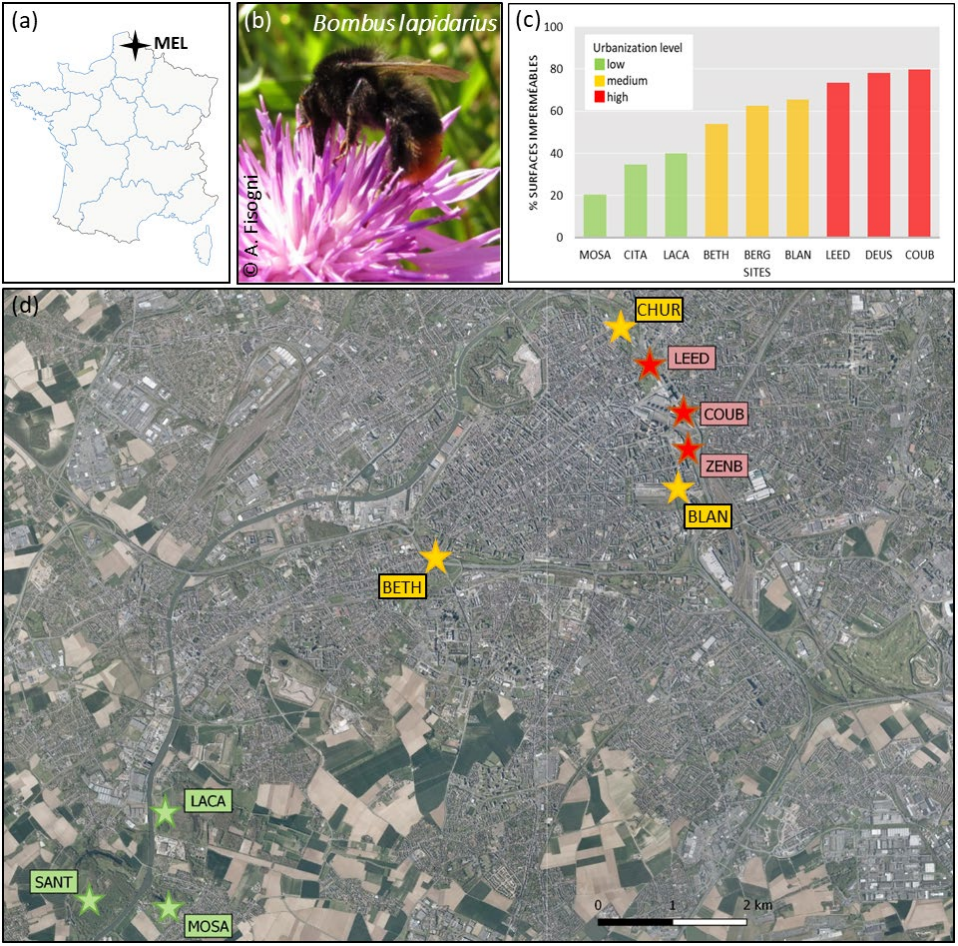
Geographic localization of the nine sites of study (d), in the Métropole Européenne de Lille (MEL), in the North of France (a). *Bombus lapidarius* workers (b) were sampled in July 2017 in all sites. Sites are located along a gradient of urbanization, defined according to the percentage of impervious surface area in a 500‐m buffer (c).

Sites were distributed along an urbanization gradient, established according to the proportion of impervious surfaces in a 500 m buffer zone around the site edges. We considered sites with less than 50% cover of impervious surfaces as low urbanization sites (SANT, MOSA, LACA; mean impervious surface ± SD = 23.0 ± 12.9, Figure 1) and sites with more than 70% cover of impervious surfaces as highly urbanized sites (LEED, COUB, ZENB; mean impervious surface ± SD = 78.0 ± 3.1, Figure 1). Sites with 50% to 69% impervious surfaces were considered as the medium urbanization level (BETH, BLAN, CHUR; mean impervious surface ± SD = 62.3 ± 5.8, Figure 1). The GPS coordinates of the selected sites are reported in Supporting Information S1: Table 1.

#### 2.1.2 Lichen Survey and Dosages

Epiphytic lichens are sentinel organisms for air quality, as they are exclusively dependent on the atmosphere for their nourishment and absorb pollutants such as metals as they do with essential elements (Cuny et al. 2018). Biomonitoring based on the analysis of corticolous epiphytic lichens was performed to assess global air quality and to generate proxy markers of long‐term exposure to nitrogen and trace elements (TEs) in the study sites. First, we estimated air eutrophication by the observation of lichen diversity and abundance for each site according to AFNOR standards (NF EN 16413; see Supporting Information for details on IBLE index calculations). High eutrophication ratio (ER) indicates a significant nitrogenous influence (from urban and/or agricultural emissions) on the lichenic flora. Second, multi‐metallic burdens were evaluated by measuring the bioaccumulation of four TEs (arsenic, cadmium, lead, and mercury) in the thalli of the foliose lichen *Xanthoria parietina* , according to AFNOR standards (NF X43–904). The measured concentrations correspond to a cumulated long‐term exposure. With an annual growth rate of approximately 1 cm, the harvesting of 6 cm diameter thalli corresponds to approximately 6 years of exposure. The sampling of lichens for the determination of TE consisted of the collection of nine small thalli that were sampled on three tree trunks (1.5 m above the soil) and pooled, except for the site of SANT, where *X. parietina* development on trees was not sufficient to get enough biomass for the analyses. In the laboratory, the lichen samples were air‐dried. Total TE concentrations were evaluated by a COFRAC‐accredited analytical laboratory, through inductively coupled plasma mass spectrometry. A composite index (mean impregnation ratio [MIR]) was finally generated to assess the level of multimetallic contamination at each site. The ratio of the measures to the background concentrations reported in the literature (respectively 0.5 μg/g, 0.2 μg/g, 15 μg/g and 0.2 μg/g for arsenic, cadmium, lead, and mercury) was calculated, and MIR corresponds to the mean value of the four ratios. Low MIR values indicate low levels of metal impregnation in the air (Occelli et al. 2016).

#### 2.1.3 Bee Survey

The local epidemic risk was considered through the abundance of *B. lapidarius* and *A. mellifera* , an abundant co‐host of pathogens found in BBs (Tehel, Brown, and Paxton 2016). Both species were surveyed in each site during the month preceding BB samplings for immune measures. Each site was visited twice between June 5 and June 29, 2017, with 13–15 days between the two visits. At each visit, bees were sampled using pan traps (deployed for at least 6 h; median: 7 h) and hand nets (variable transects across the site, by two operators for 45 min between 11 a.m. and 3 p.m.; details on sampling methods in Fisogni et al. 2020). Abundances reported here are a mean of values obtained on the two dates.

### 2.2 Collection and Preparation of BB Samples

Workers of *B. lapidarius* were collected between June 30 and July 7, 2017. At each site, seven individuals were caught with hand nets on the same day and immediately put in an icebox to be anesthetized with cold. The posterior part of the guts was removed by gently pulling out the sting and the last sternite. Bees were then quickly immersed in RNA later and flash‐frozen in liquid nitrogen. Once returned to the laboratory, samples were stored at −80°C until further processing.

Individual bees were transferred to 1 mL of Qiazol (Qiagen) for total RNA extraction. Tissues were homogenized in Qiazol by Ultra‐Turrax grinding and then processed according to the manufacturer's instructions. Genomic DNA was removed from total RNA samples by DNase I treatment (RQ1 RNase‐free DNase, Promega). For each sample, cDNA synthesis was performed from 500 ng of DNase‐treated total RNA using the RevertAid First Strand cDNA Synthesis Kit (Thermo Fisher).

### 2.3 Individual Level of Infection by Four Common Pathogens

We surveyed by PCR the presence and load of four pathogens commonly encountered in natural populations of BB (Graystock, Goulson, and Hughes 2014; McMahon et al. 2015; Tommasi et al. 2023): the neogregarine *Apicystis bombi*; two BB viruses, namely, slow bee paralysis virus (SBPV) and acute bee paralysis virus (ABPV); and a honeybee virus, deformed wing virus (DWV, using primers detecting both strains A and B).

Separate amplifications were performed for each pathogen, with the specific primers specified in Supporting Information S1: Table 2. PCR reactions were conducted with a common reaction mix for all tested individuals. The amplification of the housekeeping gene *rpl13* was performed on the same samples to control for PCR efficiency and to normalize the results. Reactions were run on a MasterCycler—Pro S (Eppendorf), with the following cycling conditions: 94°C for 5 min (1 cycle); 94°C for 15 s, Ta (°C) for 30 s, and 72°C for 1 min (35 cycles); and 72°C for 7 min (1 cycle). The PCR products were loaded onto ethidium bromide‐stained 1% agarose gels in TAE. All PCR products targeting the same target were loaded onto the same gel, together with a 1‐kbp DNA ladder molecular weight marker (100 pb DNA ladder plus; Euromedex) to confirm the expected molecular weight of the amplification products. Images of the gels after migration were acquired with an E‐Box camera (Vilber), and quantification of the band intensity was performed using the Bio‐Vision software (Vilber). The band intensity ratio between the pathogen's gene amplification and *rpl13* amplification was calculated to normalize the results. This ratio is referred to as the “relative load” of the targeted pathogen in the rest of the text.

### 2.4 Immune Gene Selection

To test for the effect of urbanization on BB health, we selected 17 genes for expression analysis using quantitative RT‐PCR. These genes were previously identified as part of a core set of genes involved in the transcriptional response of honeybees against diverse pathogens (Doublet et al. 2017) and will be used here as potential markers of immune activation in *B. lapidarius* . As markers of the humoral immune response, we selected the peptidoglycan recognition protein (PGRP) S1, the NF‐κB transcription factor Relish of the IMD immune signaling pathway, and five antimicrobial effectors: abaecin, apidaedin, defensin‐1, hymenoptaecin, and lysozyme. As markers of the cellular immune response, we used prophenoloxidase (ProPO), an enzyme involved in encapsulation, and serpin‐5, a regulator of the immune response. In addition, we included other genes associated with the response of honeybees to infection and identified as potential regulators of the immune response (Doublet et al. 2017): apolipophorin III, the heat shock proteins protein lethal(2) essential for life‐like (LOC724367) and hsp90, the enzyme AADAT, L‐LDH, glucose oxidase, and the major royal jelly protein (single locus in *Bombus terrestris* , Kupke et al. 2012). We also used Vitellogenin, an egg‐yolk precursor also involved in caste‐related division of labor in social insects that was shown to be induced by inflammatory and immune responses in honeybees (Salmela, Amdam, and Freitak 2015).

### 2.5 Immune Gene Expression

Gene choice was based on a meta‐analysis conducted on honeybees but primers were all either taken or designed from *B. lapidarius* ortholog genes' sequences. Primers for the 17 genes of interest and two reference genes selected for their expression stability (arginine kinase [AK] and actin 5C) were designed based on the corresponding sequences available in public databases. When no specific sequence was available, orthologs from close species were used. For each couple of primers, specificity was checked by sequencing the amplification product, and the amplification efficacy was estimated. The sequence of primers and corresponding efficacy are reported in Supporting Information S1: Table 3. Reactions were run on a LightCycler 480 (Roche), using iTaq Universal SYBR Green Supermix (Bio‐Rad) with the following cycling conditions: 95°C for 30 s (1 cycle), 95°C for 15 s, and 59°C for 45 s (40 cycles). A single fluorescence read was taken at the end of each 59°C step, and a sample was considered positive if the Ct value was less than 35 cycles. A melting curve was realized at the end of each PCR program to check for single peak amplification. Ratios of expression (hereafter called ‘Ct ratio’) between the gene of interest and the two reference genes were calculated using the second derivative method with the Roche LightCycler 480 software (v 1.5.1).

Immune gene expression and detection and quantification of pathogens were made on the same samples, to individually correlate the expression level of the different immune genes to the load of each of the four pathogens considered.

### 2.6 Statistics

We performed all statistical analyses in R version 4.1.1 (R Core Team 2021).

Spearman rank correlation tests (Hmisc R package, Harrell and Dupont 2022) were used to assess correlations between the biotic (eutrophic ratio; abundances of *B. lapidarius* and *A. mellifera* ) and abiotic parameters (MIR; rate of impervious surfaces) measured in the study sites, among pathogens' relative infectious loads, and among gene expression levels. The effect of the urbanization level on site parameters and prevalence of pathogens was assessed by Kruskal–Wallis tests (dunn.test R package, Dinno 2017).

To evaluate changes in pathogen loads in relation to biotic and abiotic factors, we fitted zero‐inflated GLMMs with gamma error distribution and log‐link function (glmmTMB R package, Brooks et al. 2017) using *Apicystis*, ABPV or SBPV relative loads as response variables, and urban level, BB abundance, honeybee abundance, and MIR as predictors. We excluded ER because it was highly correlated with the urbanization level (variance inflation factor > 5), and the model including urbanization level had the lowest AICc (Burnham and Anderson 2002).

To visualize correlations between variables of the same kind, principal component analyses were performed separately on pathogen loads and on gene expression data, with the FactoMineR package (Husson et al. 2022) and the factoextra R package (Kassambara and Mundt 2020). Pathogen loads (centered and scaled relative loads) or gene expression values (centered and scaled Ct ratio) were used in their respective PCA as active elements, whereas other relevant variables were included as qualitative or quantitative illustrative elements (i.e., not participating in the segregation of individuals and thus in the construction of the axes) to determine which of them may covary with either pathogen infection or specific (group of) gene(s). For each axis, the *dimdesc* function returns a correlation coefficient (for continuous variables) or an estimate (for categorical variables), both associated with a p‐value, that indicates which illustrative variables are best described by the axis, allowing to pinpoint possible correlations between our different categories of variables.

To study these correlations in more details and identify which genes covary with which local pressure, we performed partial least square (PLS) analysis (canonical mode) between the gene expression dataset (17 genes) and a dataset collecting all environmental and infection data (six continuous variables: local rate of impervious surface, MIR, ER, loads of *Apicystis*, SBPV, and ABPV), as they were collected on the same set of individuals. The canonical mode was chosen to consider the possible reciprocity of effects between gene expression and pathogen loads (Lê Cao et al. 2009). PLS identifies combinations of explanatory variables explaining maximum variation in a complex biological response, such as modulations in the expression of groups of genes, and is also well adapted in case of multicollinearity among explanatory variables (Mehmood and Ahmed 2016). The analysis was implemented with the mixOmics R package version 6.28.0 (Rohart et al. 2017). The number of components to be chosen was estimated with the *perf* function, with 10‐fold cross‐validation and 100 response permutation tests. Model quality was evaluated using the model response ranking test plot (Q2 total value), which depicts visually which components are likely to improve the model and thus help to fix the number of components to consider as predictive. Following the PLS results, we evaluated the strength of the pinpointed correlations at the level of the group of genes by PERMANOVA analyses. The Ct ratios of selected genes were used as response variables using the *adonis2* function within the R package vegan version 2.6–6.1 (Oksanen et al. 2022) with 10,000 permutations. Equal multivariate dispersions of the compared groups were checked using the *betadisper* function from the same package. P‐values were corrected for multiple comparisons using the Benjamini‐Hochberg's method to control the false discovery rate. The correlations were finally evaluated at the individual gene level by fitting separate GLMMs with gamma error distribution and log‐link function. Gene expression was used as a response variable, and the three pathogen loads including all two‐way interactions and the three‐way interactions, as well as the urbanization level, were used as predictors. Pathogen loads were centered and scaled around the mean to allow better estimations. Site identity was used as random effect in all models. Multicollinearity was checked using the R package performance (Lüdecke et al. 2021). Model assumptions were checked using the R package DHARMa (Hartig 2022).

## 3 Results

### 3.1 Site Characterization

**FIGURE 2:**
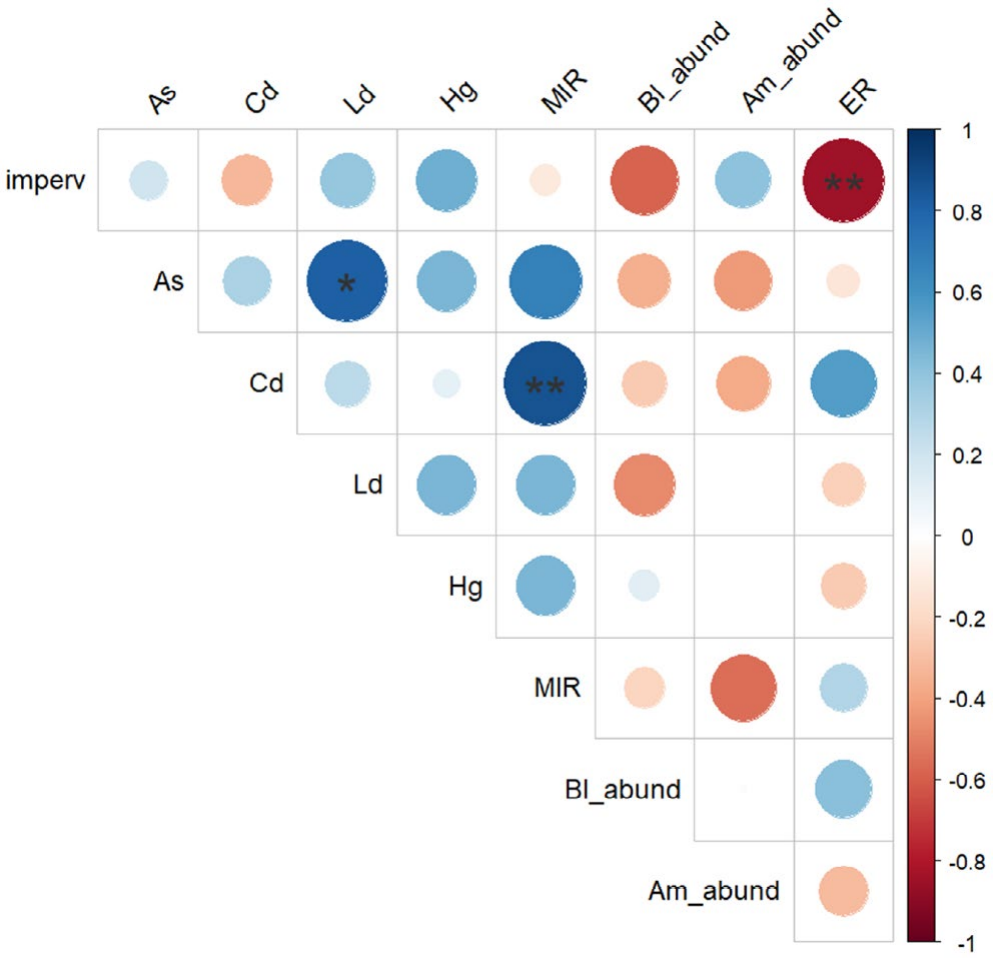
Matrix of correlations between several biotic and abiotic parameters measured in the nine sites of the study. Colors refer to the heatmap on the right and give the value of corresponding Spearman's correlation coefficients (rho). Circle size reflects the absolute value of the coefficients. Asterisks indicate significant correlations (*p < 0.05; **p < 0.01). Am_abund = *Apis mellifera* abundance; As = arsenic; Bl_abund = *B. lapidarius* abundance; Cd = cadmium; ER = eutrophic ratio; Hg = mercury; imperv = proportion of impervious surfaces; Ld = lead; MIR = mean impregnation ratio.

The results of the geographical, bee, and lichen surveys for the nine sites are summarized in Table 1. Neither honeybee nor BB abundance was significantly correlated with the level of urbanization (Supporting Information S1: Table 4), and they were not correlated with each other (Figure 2; details in Supporting Information S1: Table 5). Only *B. lapidarius* abundance tended to be negatively correlated with the percentage of impervious surface (*ρ* = 0.59, *p* = 0.092). The study of lichen communities did not reveal statistically significant differences between high‐, medium‐, and low‐urbanized sites (Supporting Information S1: Table 4). Only the ER value tended to be affected by the level of urbanization (Supporting Information S1: Table 4): the eutrophic ratio was highest in low‐urbanized sites (Table 1) and negatively correlated to the urbanization level (Figure 2; Supporting Information S1: Table 5). TE concentrations measured in lichen thalli were mostly below or close to background values, except for the site BETH (medium urbanization), where As and Cd concentrations were twice as high and Pb concentrations were 1.5 times higher than background values, and for site MOSA (low urbanization), where Cd concentrations were twice as high as background values (Table 1). TE contaminations and the MIR index were not associated with the level of urbanization (Supporting Information S1: Table 4).

**TABLE 1:** Characteristics of the sites of study.

Site	Urban survey		Bee survey		Lichen survey and dosages					
	Proportion of impervious surfaces	Level of urbanization	*B. lapidarius* abundance	*A. mellifera* abundance	Eutrophic ratio	Arsenic (mg/kg DM)	Cadmium (mg/kg DM)	Lead (mg/kg DM)	Mercury (mg/kg DM)	Mean impregnation ratio (MIR)
ZENB	0.805	High	10.5	11	3.76	0.524	0.255	7.65	0.109	0.84
COUB	0.797	High	7	6	4.07	0.568	0.169	7.21	0.107	0.75
LEED	0.736	High	21.5	6.5	3.58	0.519	0.094	5.06	0.111	0.60
CHUR	0.67	Med	10.5	15.5	4.4	0.282	0.18	6.03	0.101	0.59
BLAN	0.656	Med	5	5.5	3.94	0.589	0.193	9.8	0.079	0.80
BETH	0.541	Med	16.5	9	4.62	1.15	0.455	24.13	0.175	1.76
LACA	0.399	Low	22.5	19.5	4.55	0.356	0.162	3.47	0.071	0.53
MOSA	0.206	Low	14	4	5.03	0.41	0.478	2.22	0.095	0.96
SANT	0.086	Low	23	0.5	5	—	—	—	—	—
Background values for TE (mg/kg)						0.5	0.2	15	0.2	

*Note:* Abundances correspond to the mean number of captured bees during two visits of 45 min in the month that preceded collection for transcriptomic and epidemiologic studies. A high eutrophic ratio indicates a significant nitrogenous influence (from urban and/or agricultural emissions) on the lichenic flora.

Abbreviations: DM = dry matter, TEs = trace elements.

### 3.2 Prevalence of Pathogens

*Apicystis*, SBPV, and ABPV were detected at all sites, whereas DWV was not detected in any of the samples. SBPV had the highest prevalence, infecting 76.3% of the collected *B. lapidarius* specimens, against 71.9% for ABPV and 30.5% for *Apicystis*. In all, 19.3% of the sampled individuals bore all three pathogens, whereas 14.04% had none. More than half (52.63%) were infected by two pathogens, the most frequent association being ABPV + SBPV. All the individuals infected by *Apicystis* (*n* = 17) were also contaminated by one (35.3%) or two (64.7%) viruses. Only *Apicystis* prevalence and SBPV prevalence tended to be correlated with each other (*ρ* = 0.66, *p* = 0.051). Pathogen prevalence was not correlated with any of the other biotic or abiotic factors tested (Supporting Information S1: Table 5), with only a trend detected between SBPV prevalence and lead contamination (*ρ* = 0.68, *p* = 0.066). Prevalence did not significantly differ among urbanization classes for any of the three detected pathogens (Supporting Information S1: Figure 1, Table 4).

### 3.3 Pathogen Loads

**FIGURE 3:**
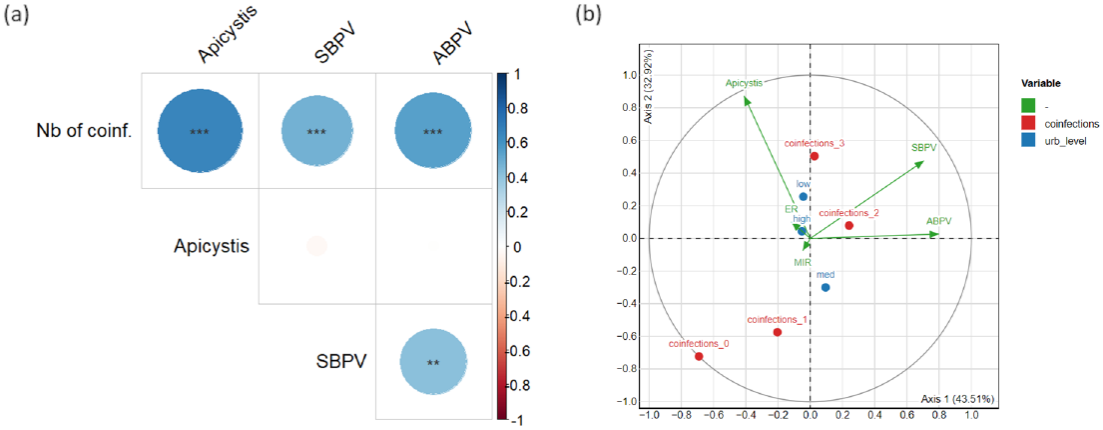
(a) Matrix of correlations between the relative loads of the three pathogens detected in *B. lapidarius* for all nine sites. Virus loads are estimated by the standardized numbers of genome copies, whereas *Apicystis* load is estimated by the standardized expression of an *Apicystis* housekeeping gene. Colors refer to the heatmap on the right and give the value of corresponding Spearman's correlation coefficients (rho); the areas of circles also reflect the absolute value of these coefficients. Asterisks indicate significant correlations (**p < 0.01; ***p < 0.001). (b) PCA correlation circle showing a projection of original variables (*Apicystis*, SBPV, and ABPV relative loads) in the principal component space. Illustrative variables are also plotted as vectors for the continuous variables (ER and MIR) and as centers of gravity for categorical variables (level of urbanization in blue and number of coinfections in red). The vector's length represents the variable's overall contribution, whereas the vector's projection onto one of the principal components gives the variable's contribution to that specific axis.

Individual relative load for each pathogen was highly correlated with the individual number of pathogens (Figure 3A and Supporting Information S1: Table 6). As for the prevalence, SBPV and ABPV relative loads were significantly correlated (Supporting Information S1: Table 6). None of the pathogen loads were significantly correlated with any of the abiotic (urbanization level, MIR) or biotic (BB or honeybee abundance) variables tested (Supporting Information S1: Table 7; see Supporting Information S1: Figure 2 for the distribution of the relative loads according to the urbanization level of the sampling site). Concerning the PCA performed on individual pathogen loads (Figure 3B), the first axis (43.5% of the total variance explained) was mainly driven by viral loads, whereas differences on the second axis (32.9% of variance explained) mainly rested upon *Apicystis* load (see Supporting Information S1: Table 8 for details). This second axis can significantly discriminate individuals according to the number of pathogens they hosted (“coinfections” variable, added here as supplementary variable; *R* ^2^ = 0.17, *p* = 0.02). None of the axes discriminated individuals according to the ER value, the MIR index, or the level of urbanization of their sampling site.

### 3.4 Immune Gene Expression

Within the 17 genes tested, three groups of genes were correlated with each other (Supporting Information S1: Figure 3; details in Supporting Information S1: Table 9). The first group corresponded to the four antimicrobial peptides (apidaecin, hymenoptaecin, abaecin, and defensin‐1). The second group included PGRP‐S1, serpin‐5, HSP90, apolipophorin III, lysozyme, protein lethal essential for life‐like, and relish. The third group gathered AADAT, L‐LDH, and MRJP. Abaecin and L‐LDH are also individually correlated to many genes that belong to the two other groups.

**FIGURE 4:**
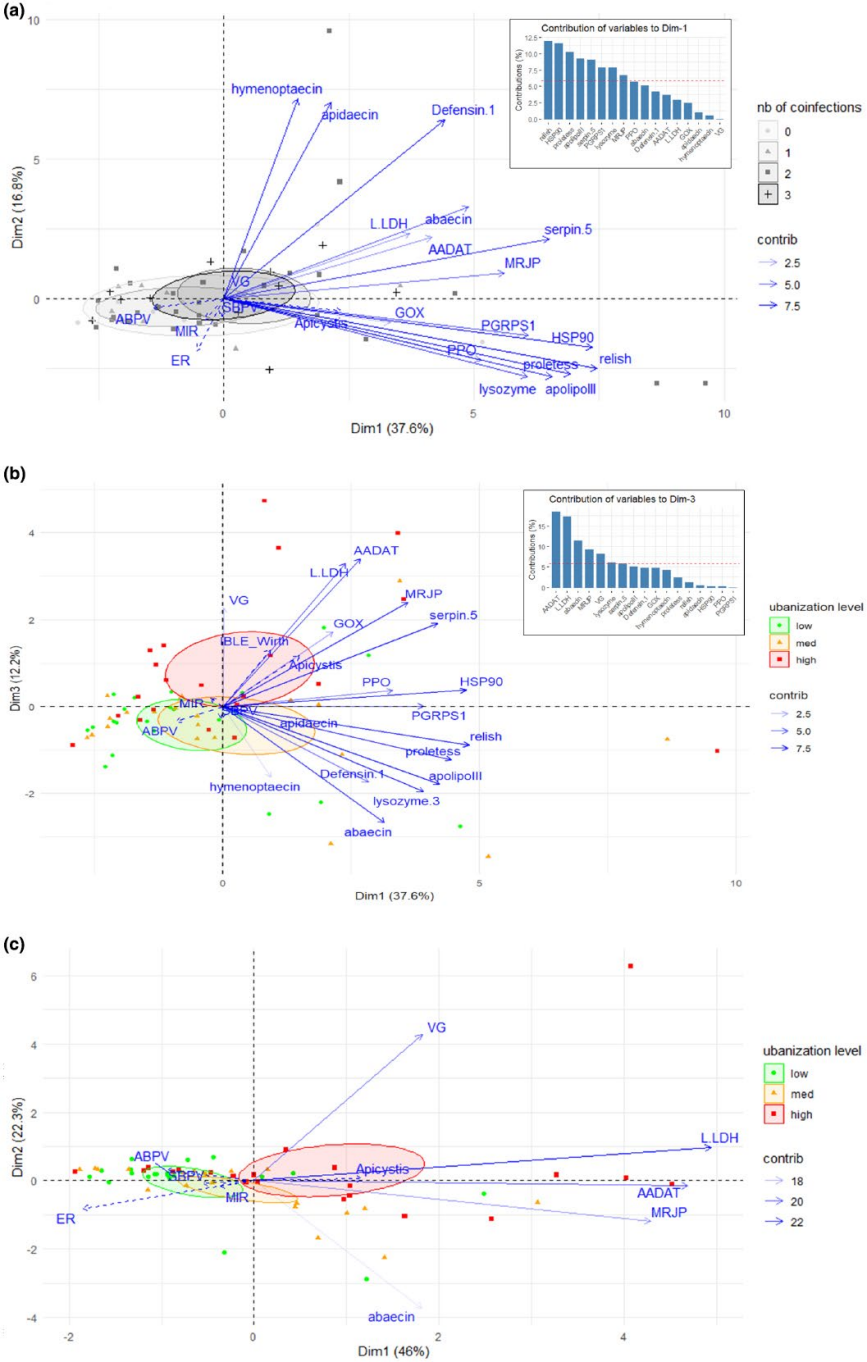
(a) Principal component analysis (PCA) biplot ordering the expression of the 17 immune genes considered. Results displayed correspond to projections along the first and second dimensions (variance explained in brackets). The variables that significantly contribute to the first axis (Dim1) are those higher than the red dashed line in the contribution graph (A, insert; the red dashed line indicates the expected average contribution). Confidence ellipses gather individuals along their level of infections (0, 1, 2, or 3 pathogens detected). (b) Same PCA biplot as in A, with projections along the first and third dimensions. The variables significantly contributing to the third axis are those higher than the red dashed line in the contribution graph (B, insert). Confidence ellipses gather individuals relative to the level of urbanization of their sampling site (low in green, medium in yellow, and high in red). (c) PCA biplot ordering the expression of five genes (AADAT, L‐LDH, abaecin, MRJP, vg), selected as they contribute significantly to the third axis in (B). Confidence ellipses gather individuals relative to the level of urbanization of their sampling site (low in green, medium in yellow, and high in red).

In the PCA performed to visualize the relationships between the local pressures (site characteristics or relative infectious loads) and the expression of the targeted immune genes, the first axis explained 37.6% of the total variance, overall separating individuals with high levels of gene expression from those with low levels (Figure 4A). Individuals that carry more pathogens (ellipses in gray gradient) segregated toward higher levels of expression along Dim1 (“coinfections” variable included as supplementary variable; Figure 4A). Among the different variables, genes belonging to the second group (relish, HSP90, protein lethal essential for life‐like, apolipophorin III, serpin‐5, PGRP S1, and lysozyme; Supporting Information S1: Figure 3), together with MRJP, contributed the most and significantly to the first axis, each showing more than 65% of correlation with the axis (Figure 4A, insert; see Supporting Information S1: Table 10 for extensive results of the PCA). The second axis explained 16.8% of the total variance and was defined by the first block of correlated genes: hymenoptaecin (84% of correlation), apidaecin (82%), and defensin‐1 (75%). The third axis explained 12.2% of the total variance and was notably determined by the expression of not only AADAT (62%), L‐LDH (60%), and MRJP (44%), belonging to the third block of correlated genes, but also abaecin (48%) and vitellogenin (41%) (Figure 4B, insert).

The first two PCA axes were not significantly related to any of the illustrative variables tested (ER, MIR index, pathogen loads), whereas the coordinates of the individuals along the third axis were partly correlated with the level of urbanization (*R* ^2^ = 0.18, *p* = 0.003) (Figure 4B). More precisely, individuals who have positive coordinates along Dim3 tended to come from sites with a high level of urbanization (for urbanization level = high: estimate = 0.86, *p* = 0.0005). Five genes significantly contributed to this discrimination: AADAT, L‐LDH, abaecin, MRJP, and vitellogenin (Figure 4B, insert). When considering the expression of these five genes only in a new PCA analysis, the three levels of urbanization were separated along the first axis, which explained 46% of the total variance of the individuals' coordinates (Figure 4C). The genes L‐LDH (corr. coeff. = 0.89, *p* = 3.06e‐22), AADAT (corr. coeff. = 0.84, *p* = 5.8e‐18), and MRPJ (corr. coeff. = 0.77, *p* = 1.7e‐13) contributed the most to the first axis (extensive results provided in Supporting Information S1: Table 11). These genes were overexpressed at high levels of urbanization and underexpressed at low levels of urbanization (*R* ^2^ = 0.17, *p* = 0.004; for level_urb = high: estimate = 0.84, *p* = 0.001; for level_urb = low: estimate = −0.66, *p* = 0.013).

**FIGURE 5:**
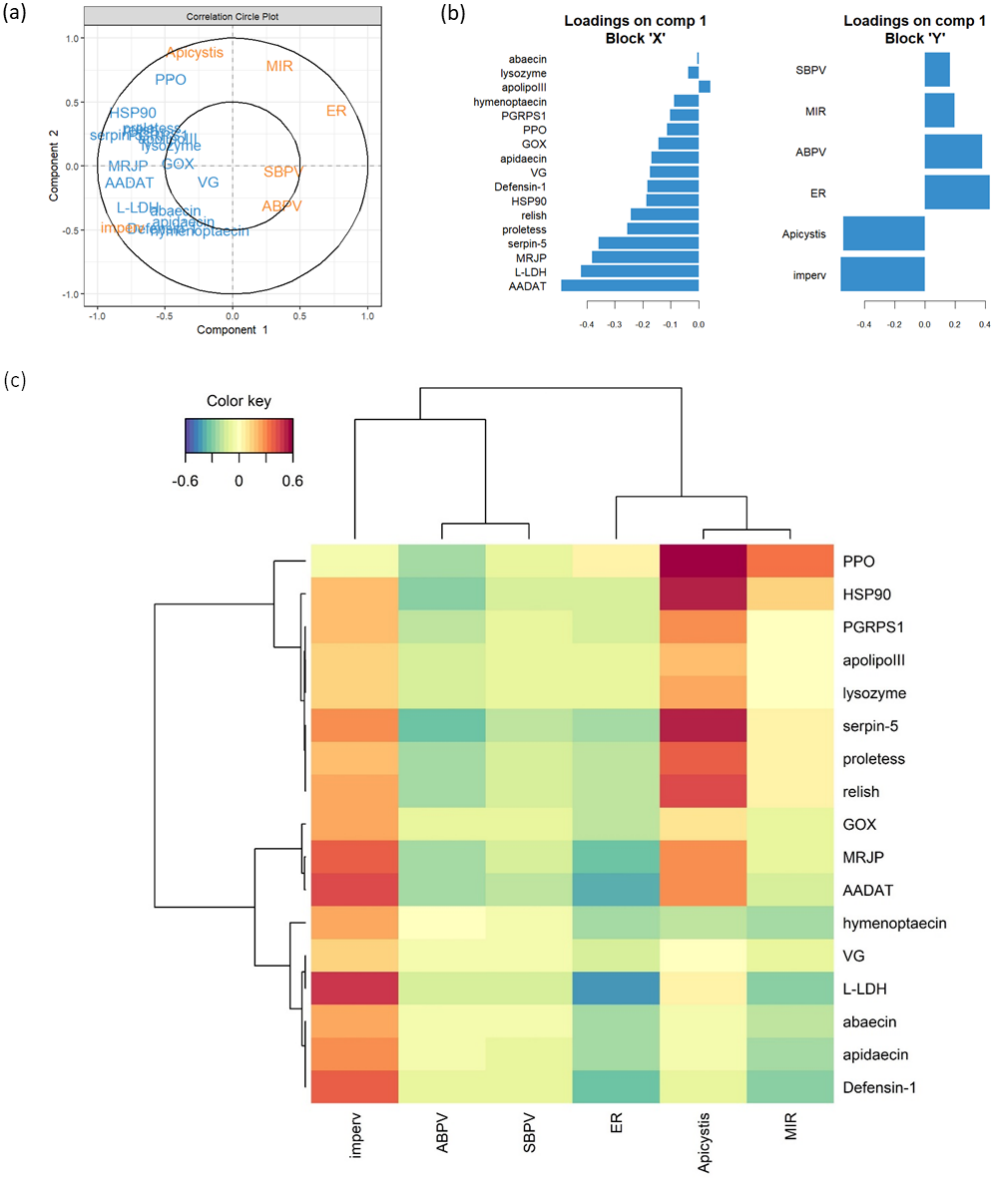
(a) Correlation circle plot for the first two PLS components. The two circumferences show correlation coefficient radii at 0.5 and 1.0. The farther from the center, the greater the association of this variable with the component. Variables projected in the same direction of the plot are positively correlated, whereas variables in diametrically opposite positions are negatively correlated. In blue: Variables from the gene expression dataset; in red: Variables from the site characteristics/pathogen loads dataset. (b) Loading plots of each variable having the maximal discrimination ability on the first component in each dataset. (c) Clustered Image Maps of the gene relative expression and local parameters in the first two PLS components. Hierarchical clustering was derived using the complete linkage method and Pearson correlation as distance. The resulting heatmap contains the genes in rows and site characteristics or pathogen loads in columns. The color key gives the value of corresponding correlation coefficient, with red indicating positive correlation and blue indicating negative correlation.

The PLS analysis retrieved components that maximize covariation between the gene expression dataset and the dataset that gathers information about site characteristics and infection loads. After cross‐validation, only the first component was retained, that is, considered good at predicting/generalizing one dataset by the other (details in Supporting Information S1: Table 12). As a consequence, the second axis was kept for graphical needs but only the first axis should be considered for interpretation. The latter explains 32.6% of the total variance of the gene expression dataset and 28.8% of the total variance of the site characteristics/infection loads dataset. We found high covariation between the rate of impervious surface and the *Apicystis* infection load on the one hand, and between the expression of AADAT, L‐LDH, MRJP, and serpin‐5 on the other (Figure 5a,b). L‐LDH, AADAT, defensin‐1, and MRJP were the most positively correlated variables with the rate of impervious surface (corr. coeff. = [0.37–0.48], Figure 5c), whereas serpin‐5, PPO, and HSP90 expression were highly positively correlated with *Apicystis* load (corr. coeff. > 0.5, Figure 5c). Overall, the different gene expressions were poorly correlated with ABPV and SBPV loads and ER and MIR values (corr. coeff. = [−0.34–0.12]), except for l‐LDH and AADAT that were negatively correlated with ER (−0.43 and −0.37, respectively; Figure 5c). All coefficients are reported in Supporting Information S1: Table 12.

Following the PLS results, we tested the multivariate effect of the urbanization level and *Apicystis* infection on different combinations of genes by PERMANOVA. When considered together, AADAT expression and L‐LDH expression were affected by the level of urbanization, and to a lesser extent by *Apicystis* infection, without significant interaction between the two factors (Table 2). Similar effects were found when including MRJP expression, with the addition of a slight interaction effect (Table 2), whereas no effects were observed when including defensin‐1 expression (Supporting Information S1: Table 13). Based on PCA observations, we also tested the previous combinations including abaecin and vitellogenin but no combination showed significant effects (Supporting Information S1: Table 13). We found a significant effect of *Apicystis* infection but not of urbanization level on the joint expression of PPO, serpin‐5, and HSP90 (Table 2).

**TABLE 2:** PERMANOVA results on the effects of urbanization level, *Apicystis* infection, and their interaction on the expression of the AADAT and L‐LDH genes alone (top) on AADAT, L‐LDH, and MRJP gene expression (middle) and on PPO, serpin‐5, and HSP90 gene expression (bottom).

Factor	df	Sum of sqs	*R* ^2^	F	Pr (>*F*)	*p* adj
Multivariate effects on AADAT and L‐LDH expression						
Urbanization level	2	44.920	0.15686	5.8286	0.0039	**0.0117**
Apicystis infection	1	20.791	0.07260	5.3954	0.0263	**0.0395**
Urb_level:Apicystis	2	16.432	0.05738	2.1321	0.1276	0.1276
Residual	53	204.235	0.71316			
Total	56	286.378	1.00000			
Multivariate effects on AADAT, L‐LDH, and MRJP expression						
Urbanization level	2	51.62	0.14017	5.3719	0.0022	**0.0066**
Apicystis infection	1	27.12	0.07365	5.6453	0.0191	**0.0287**
Urb_level:Apicystis	2	34.87	0.09469	3.6289	0.0490	**0.0490**
Residual	53	254.64	0.69148			
Total	58	368.25	1.00000			
Multivariate effects on PPO, serpin‐5, and HSP90 expression						
Urbanization level	2	2.950	0.01246	0.4485	0.7582	0.7582
Apicystis infection	1	36.333	0.15350	11.0465	0.0022	**0.0066**
Urb_level:Apicystis	2	23.085	0.09753	3.5094	0.0544	0.0816
Residual	53	174.321	0.73650			
Total	58	236.689	1.00000			

*Note:* Statistically significant effects (with BH correction) are highlighted in boldface.

Linking individual gene expression with biotic and abiotic local pressures, we observed that AADAT gene expression was significantly positively correlated with *Apicystis* load (Supporting Information S1: Figure 4A), and AADAT was more expressed with increasing *Apicystis* load when ABPV load was higher (positive two‐way interaction, Supporting Information S1: Figure 4B). Moreover, AADAT (Supporting Information S1: Figure 4C) and l‐LDH (Supporting Information S1: Figure 4D) gene expression significantly increased with increasing levels of urbanization. All model coefficients are reported in Supporting Information S1: Table 14.

## 4 Discussion

Our study is one of the few to have jointly investigated the effects of environment quality and local biotic pressures on the health status of wild bees. Our results showed no clear correlation between the level of urbanization and local air quality (neither when assessed by lichen diversity nor with thalli analysis of metal contaminants), local competition (bee abundances), or parasite pressure (prevalence and loads). We therefore faced an interesting situation, where pathogens and parasite pressures, air pollution, and local competition were not conflated with urbanization levels, allowing to test their effects on BB immune response independently. Several studies have evidenced weak effects of inbreeding on immune performance in bees (e.g., Gerloff, Ottmer, and Schmid‐Hempel 2003; Lee, Brown, and Oldroyd 2013), thus reducing the risk of gene expression changes due to inbreeding depression common in urban milieu. We therefore selected key immune genes and measured their expression in response to different local environmental and anthropogenic pressures. Two main factors appeared to be associated with the expression of the selected genes: *Apicystis* infection and the local rate of impervious surface (as a proxy for urbanization level).

Infections by SBPV and/or ABPV were the most frequent (prevalence of 76% and 71%, respectively), and the strong correlation of their respective loads in coinfection suggests a possible synergistic interaction between the two viruses (e.g., enhancement of viral replication; Du, Wang, and Zhang 2022; Goto et al. 2016) in *B. lapidarius* . However, the two virus loads were each weakly associated with variation in immune gene expression. Conversely, *Apicystis*, which is described as a common BB pathogen (Goulson, Whitehorn, and Fowley 2012), was the least frequent pathogen detected in this study (prevalence of 30.5%). Interestingly, it was never detected alone but always in association with at least one virus, which suggests a facilitating effect of viral infection on *Apicystis* infestation, although further investigation is needed to disentangle such interactions. Many genes had their expression that covaried with the *Apicystis* load, and most notably PPO, serpin‐5, and HSP90. PPO is involved in cellular immunity and contributes to the melanization cascade that ends up in the sequestration of parasites such as *Apicystis*. Serpins are widely distributed serine protease inhibitors, shown to be involved in immune pathways regulation, including melanization, in *A. mellifera* (Zou et al. 2006). The positive regulation of these two genes therefore makes sense in the context of a parasitic infection, just like that of HSP90, which is a widely recognized stress marker (Taipale, Jarosz, and Lindquist 2010; Abou‐Shaara 2024).

Three immune markers—AADAT, L‐LDH, and MRJP—jointly and positively covaried with the local rate of impervious surface or in case of *Apicystis* infection. This is also true for AADAT and L‐LDH expression levels when considered alone. We hypothesize that the effect of urbanization level and *Apicystis* infection may operate largely independently on these gene expressions, because no or very weak interaction was found between them. Moreover, the influence of urbanization level was predominant in both univariate and multivariate analyses.

We found no relationship between the urbanization level and the prevalence or load of four major bee pathogens, contrarily to a growing number of studies, predominantly on birds, that highlighted increasing pathogenic pressures with urbanization levels (Theodorou et al. 2016; Youngsteadt et al. 2015; reviewed in Minias 2023). Moreover, BB and honeybee abundances, which may be related to pathogen infection and spillover, did not increase with urbanization level either. In summary, in this study and with regard to the variables surveyed, while BBs from more urbanized areas did not seem to suffer from higher infection rate and were not exposed to poorer air quality or higher bee density, they still had a partially activated immune system, with the expression of AADAT, L‐LDH, and MRJP genes increasing with the level of urbanization.

AADAT and L‐LDH genes code for metabolic enzymes, whose immune functions are probably indirect in wild bees, as suggested in other models. In humans, AADAT is involved in the endogenous tryptophan metabolism and the kynurenine pathway, producing kynurenic acid (KYNA). This molecule has clear anti‐inflammatory effects (Marszalek‐Grabska et al. 2021), studied notably in the inflammatory bowel disease in which the protective effect of KYNA involves rewiring of cellular energy metabolism (Michaudel et al. 2023). Although so far not linked to insect immune processes, the kynurenic pathway has been characterized in insects, including honeybees (Linzen 1974; Smirnov et al. 2006). Noteworthy, AADAT was shown to be involved in trans‐generational immune priming in *B. terrestris* (Barribeau, Schmid‐Hempel, and Sadd 2016). In turn, L‐LDH activation is clearly associated with the metabolic changes that occur in inflammatory immune cells upon their activation, as evidenced in vertebrate (Escoll and Buchrieser 2018; Soto‐Heredero et al. 2020) and in insect cells (Dolezal et al. 2019). Indeed, in activated immune cells, ATP and NADH production relies more on aerobic glycolysis (low output but great rapidity) than on mitochondrial oxidative phosphorylation (more efficient but too slow). l‐LDH then has the major role of regenerating NAD+ from NADH by converting pyruvate to lactate (aerobic glycolysis), thus supplementing the broken TCA cycle and the suppressed oxidative phosphorylation steps, in what is called the Warburg effect (Escoll and Buchrieser 2018; Palsson‐McDermott and O'Neill 2013; Dolezal et al. 2019). Accordingly, higher L‐LDH expression has been commonly reported in activated hemocytes or infected tissues of various insects (fruit flies, Johansson, Metzendorf, and Soderhall 2005; mosquito, Pinto et al. 2009; moth, Shelby and Popham 2012) or associated with higher resistance to parasites (Oliveira et al. 2011).

MRJP is a protein homologous to BtRJPL in *B. terrestris* and to MRJP9 in *A. mellifera* (Kupke et al. 2012). These MRJP homologs, contrarily to the other honeybee MRJPs (*mrjp*1‐8), are not thought to contribute to larval feeding, but were rather proposed to fulfill a wider physiological role, such as a primitive role in food digestion or modification (Albert et al. 2014; Buttstedt, Moritz, and Erler 2013). Moreover, decreased levels of BtRJPL were associated with lower metabolic activity in *B. terrestris* (Kupke et al. 2012). L‐LDH, AADAT, and, more hypothetically, MRJP therefore may have an immune role rooted in the metabolic pathways that underlie immune functions, and are likely among the key regulators that orchestrate energetic trade‐offs between immunity and other physiological functions. Accordingly, AADAT and L‐LDH were shown to be the most interconnected genes among those consistently modulated in response to various pathogens in *A. mellifera* , designating them as upstream genes in the gene cascade that regulates immune responses (Doublet et al. 2017). Even more than the other genes investigated here, their upregulation could root any immune activation, whatever the trigger and perhaps beyond the infectious causes. Because immune processes are energetically costly, the organism whose immune system is triggered, whatever the cause (i.e., infection or dysregulation), must reallocate mobilizable energy toward immune functions (e.g., Lochmiller and Deerenberg 2000; Ashley, Weil, and Nelson 2012) and proceed to a switch in cell metabolism aimed at rapidly producing ATP and immune effectors (Escoll and Buchrieser 2018). This Warburg effect also operates in insects and constitutes a very early step in any activation of the immune system (Dolezal et al. 2019). In the present study, individuals living in more urbanized areas exhibited an upregulation of L‐LDH, AADAT, and MRPJ, which could constitute a shift in their immunometabolism. This shift does not appear here to be associated with higher infection risk or poorer air quality. Beyond infection risk and air quality, urban environment can be the source of many other stresses for wild species, related to poor nutritional quality of urban food (Cummings et al. 2020), multiple exposures to various pollutants (chemicals, noise, artificial light), high temperatures (i.e., heat island effect, Hemberger, Rosenberger, and Williams 2023; Kuo et al. 2023), or reduced habitat quality and accessibility (Persson et al. 2020). The effects of urban environments on invertebrate organisms are poorly documented, but it is possible that all the negative influences mentioned above collectively constitute chronic stress (i.e., long‐term or repeated exposure to adverse stimuli), which can increase basal levels of stress markers such as octopamine in insects (Adamo and Baker 2011; Rech et al. 2022). The tight links that exist between the endocrine/neuroendocrine systems and the immune system could then lead to a modification of basal immune functions (Adamo 2012, 2017). Likewise, in blue tits, genes associated with inflammation appeared upregulated in urban compared to that in forest populations, without any link with the local prevalence of different pathogens (Capilla‐Lasheras et al. 2017). A mechanistic explanation may be emerging, with bird populations living in cities presenting different DNA methylation profiles from those living in natural environments (Caizergues et al. 2022). In the case of BB and considering the correlations evidenced in the present study, we propose that the expression level of L‐LDH and AADAT (and to a lesser extent MRJP) could be used as biological markers to detect wild bees facing high urban stresses and suggest more thorough studies. Future research should aim to determine whether the shift in immunometabolism is adaptive in *B. lapidarius* , preparing the organism to fuel future immune activation, or rather the hallmark of an immune dysregulation. In a genome‐wide study on the same species collected in nine German cities, Theodorou et al. (2018) evidenced only low genetic differentiation among sites, but nevertheless identified several loci under directional selection that were associated with surrounding impervious surface level, leading them to suggest a possible adaptation of *B. lapidarius* populations to urbanization. The pinpointed genes did not include ADAAT or L‐LDH but were largely involved in metabolic processes, highlighting the pressure exerted by urbanization on this specific aspect of homeostatic regulations.

Alternatively, the specificity of the resources acquired in highly urbanized areas is a possible cause of the shift in BB immunometabolism, because tight links exist between nutrition and immunity (Minias 2023). If cities can offer to pollinators a non‐negligible flower cover compared to rural counterparts (Baldock et al. 2015, 2019), food resources may nevertheless be less abundant in highly urbanized areas compared to those in areas of lower urbanization level, notably because of less and smaller green cover areas (Theodorou et al. 2020). They may also be more difficult to access because of higher fragmentation (which profoundly affects large pollinators with high requirements, Zaninotto, Fauviau, and Dajoz 2023) and perhaps of lower quality (e.g., lower content of chlorophyll or delays in phenology, de Barros Ruas, Costa, and Bered 2022). In our case, BB had access to a similar diversity of plants at all sites (Fisogni et al. 2020) but could be malnourished in the city center where many bees concentrate on small areas (cf. honey‐pot effect, Theodorou et al. 2020) of perhaps less rewarding and/or less nourishing floral resources as discussed above. Moreover, land use affects the composition of secondary metabolites in floral rewards, which are known to play a significant role in the diet of pollinators, acting as antinutrients, essential nutrients, or nutraceuticals for BBs (Pioltelli et al. 2024; Sculfort et al. 2021). BBs may consequently have their physiology more affected (i.e., shifted immunometabolism) compared to less stressed conspecifics, especially suffering more from their pathogen burden. Indeed, in our study, SBPV and ABPV prevalence was high (> 70% for at least one of the two viruses) throughout the sites studied, and the coinfection by both viruses was the most frequent situation. Yet, it has been shown that viral virulence was condition dependent in BBs, with, for instance, SBPV inducing higher mortality in *B. terrestris* submitted to starvation (Manley, Boots, and Wilfert 2017). Access to poor quality resources in highly urbanized sites could lead local populations to tolerate their viral load less well, triggering immune changes such as immunometabolism shift.

Whatever its cause, the metabolic shift toward immune activation in the most urban BBs may represent a cost that exerts an additional burden on already fragile pollinator populations. This cost could correspond to a monopolization of energy resources by the immune system to the detriment of other functions. Indeed, as an essential life‐preserving process, the immune response can override less important physiological processes (e.g., growth or reproduction) when energy must be shared, a privilege status summed up in the concept of the “selfish immune system” (Dolezal et al. 2019; Straub 2014). For example, higher immune gene expression was associated with lower reproductive success in urban blue tits (Capilla‐Lasheras et al. 2017). Other costs can also arise from side effects due to the increased expression of the concerned immune genes. Such side effects of immune gene expression have already been evidenced in bees: Mobley and Gegear (2018) showed that an induced chronic high expression of antimicrobial peptides impairs short‐term memory in honeybees, reducing cognitive performance and foraging efficiency. Further investigations will be needed to fully understand the consequences of the metabolic status observed in pollinators living in highly urbanized contexts. If urban areas can act as refuges for pollinators as suggested (Baldock et al. 2015, 2019; Theodorou et al. 2020), and in a context of increasing urbanization, it appears necessary to deepen our knowledge on the physiological aspects of urban life for insects to better evaluate their health, at the individual and population scales, in this specific environment.

## Author Contributions

**Virginie Cuvillier‐Hot:** conceptualization (lead), data curation (lead), formal analysis (lead), investigation (equal), methodology (lead), project administration (lead), writing – original draft (lead). **Alessandro Fisogni:** formal analysis (equal), methodology (equal), writing – review and editing (supporting). **Vincent Doublet:** conceptualization (equal), writing – review and editing (supporting). **Sylvain Guillot:** investigation (equal). **Anne‐Catherine Holl:** investigation (equal). **Julie Leclercq‐Dransart:** investigation (equal), writing – review and editing (equal). **Florent Occelli:** investigation (equal), writing – review and editing (equal). **Yves Piquot:** conceptualization (supporting), project administration (equal), writing – review and editing (equal). **Nina Hautekèete:** conceptualization (equal), project administration (equal), writing – review and editing (equal).

## Conflicts of Interest

The authors declare no conflicts of interest.

## Supporting information


Data S1.

## Data Availability

Our data will be archived in a Zenodo Digital Repository (http://doi.org/10.5281/zenodo.10606744) should the manuscript be accepted for publication.
