# Supplementary material for: Urbanization Shifts Immunometabolism in a Common Bumblebee
Source: Ecol Evol. 2024 Dec 22;14(12):e70743. doi: 10.1002/ece3.70743 (PMC11663479; doi:10.1002/ece3.70743)
Supplement: Supplementary file 1 — Data S1. [file ECE3-14-e70743-s001.docx]

**SUPPLEMENTARY MATERIALS**

**Table S1.** GPS coordinates of the 9 sites of study

| **Site** | **Municipality** | **Latitude** | **Longitude** |
| --- | --- | --- | --- |
| ZENB | Lille | 50°37'54.038'' N | 003°04'50.581'' E |
| COUB | Lille | 50°38'08.299'' N | 003°04'48.373'' E |
| LEED | Lille | 50°38'32.074'' N | 003°04'24.741'' E |
| CHUR | Lille | 50°38'48.717'' N | 003°04'02.861'' E |
| BLAN | Lille | 50°37'35.958'' N | 003°04'42.783'' E |
| BETH | Lille | 50°37'5.261'' N | 003°01'57.462'' E |
| LACA | Haubourdin | 50°35'11.814'' N | 002°58'49.077'' E |
| MOSA | Houplin-Ancoisne | 50°34'28.096'' N | 002°58'56.301'' E |
| SANT | Santes | 50°34'32.481'' N | 002°58'01.114'' E |

**Eutrophic ratio (ER) calculation**

According to AFNOR standards (NF EN 16413), a high eutrophication ratio (ER) indicates a significant nitrogenous influence (from urban and/or agricultural emissions) on the lichenic flora. Here we detailed its calculation.

Step 1 - Lichen observation

For each station, we selected 3 trees of diverse species if possible. To avoid specific ecological conditions favoring the presence of certain lichen species, the selected trees must meet the following validity criteria: minimum circumference of 40-60 cm, tree inclination < 10 degrees, non-branched tree at the base, and undamaged bark. Epiphytic lichen communities are identified using a standardized grid measuring 10 cm in width and 50 cm in height, divided into 5 squares of 10x10 cm. This grid is placed on the trunk at the four cardinal points, with its base located 1 m above the ground. For each cardinal orientation, the lichen species present are listed, and the frequency of each species (noted as F, ranging from 0 to 5) is assessed. Then, the coverage (C) of each species is evaluated in each grid square using the following code: 1 (≤5%), 2 (5-25%), 3 (26-50%), 4 (51-75%), 5 (≥75%). The average coverage is then calculated for each species present in the observation grid.

Step 2 - Epiphytic Lichen Biological Index (IBLE) calculation

IBLE calculation is performed based on the product P of the frequency and coverage of taxa: P = C x F, where C is the average coverage of species in the grid and F is the frequency of species in the grid. This calculation is obtained for each species and each cardinal orientation of a tree. The average of P is calculated for each cardinal orientation of the station. IBLE corresponds to the sum of the average P for all lichen species at the station.

$$IBLE=mean P\left( North \right)+mean P\left( South \right)+mean P\left( East \right)+mean P\left( West \right)$$

IBLE is a combination taking into account the frequency and coverage of each observed lichen species per station. Thus, for a same value, it is possible to conclude:

- low lichen diversity and low coverage;
- low lichen diversity and high coverage;
- high lichen diversity and low coverage;
- high lichen diversity and high coverage.

Step 3 – Weighted IBLE calculation (IBLE_Wirth_)

This index corresponds to a phytosociological score and cannot directly lead to a classification of the overall air quality level. Given the nitrophilic influence of the environment, the IBLE value is then weighted by the Wirth index (Wirth, 2010), an ecological index expressing the ecological amplitude of a species in relation to environmental factors, here eutrophication.

Step 4 – Eutrophic ratio calculation

The eutrophic ratio is finally calculated by dividing weighted IBLE by IBLE (ER = IBLE_Wirth_/IBLE).

**Table S2.** Primers used to detect and quantify infection by 4 common BB pathogens

| **Target** | **Forward primer 5’-3’** | **Reverse primer 5’-3’** | **Ta (°C)** | **Amplicon size (bp)** |
| --- | --- | --- | --- | --- |
| *A. bombi* 18S | TGATCCATAATAATTTTGTGAATCGCG | AGTGCTATGTTTGTTTTTAACGACA | 60.7 | 392 |
| SBPV | GAGATGGATMGRCCTGAAGG | CATGAGCCCAKGARTGTGAA | 55 | 915 |
| ABPV | CYATGGACACACCCTATGTG | CGCCATTTTGGTACTTCTCC | 55 | 1034 |
| DWV | AACTGGCGAYCATACTCAGC | WCCAGGCACMCCACATACAG | 62 | 644 |
| *rpl13* | GCCTATTGTTCATTGCCCAAC | TAGTCTGGCATCAGCCCTAGC | 61.5 | 409 |

Primer sequences were drawn from the literature for SBPV (Manley et al., 2017)^[[1]](#footnote-1)^ and DWV (Wilfert et al., 2016)^[[2]](#footnote-2)^

**Table S3.** Primers used to quantify immune gene expression. Genes used as references are in gray background.

| **Gene** | **Source sequence** | **Forward primer 5’-3’** | **Reverse primer 5’-3’** | **size (bp)** | **Efficiency** |
| --- | --- | --- | --- | --- | --- |
| **AADAT**  kynurenine/alpha-aminoadipate aminotransferase | Bi: XM_003487614.3 | AATTGGGTTCAGCTTTGCAG | GAGACGATCACGATGTCACG | 127 | 1.83 |
| **Abaecin** | Bl: KC662132.1 | GCGCATCTTTTCTTTTCGTC | CACGAGTTCTCTATTAAAGGCAAA | 97 | 1.90 |
| **Apidaecin** | Bi: XM_012389042.1^1^ | TCTACCACCACAATCCCAAATACA | AATTGGTGGGAGACTTATAGGTCG | 114 | 1.94 |
| **Apolipophorin III** | Bt: XM_012318967.1 | AACAGAGCACCAACTTCATTACC | CTTAACGCCGTCCCATAGTC | 107 | 1.91 |
| **Defensin-1** | Bl: KC662144.1 | CTTCTCTCCATCAAAGGAGTCG | TCACGTACTTGCGACAAAGG | 115 | 1.91 |
| **GOX**  Glucose oxidase | Bi: XM_003491313.2 | GAATGTACACCCAACGAGCA | TCGGTCCCGAAATCGTTA | 93 | 2.02 |
| **HSP90** | Bt: XM_003396849.2 | TGTCGTCCAACAACTGAAAGA | CTTTGTCTTCTTCGCGCTTC | 113 | 1.81 |
| **Hymenoptaecin** | Bl: KX146600.1 | TCCTCGTTTTCGACGACAC | TAATCGACGTCCAAGGATGG | 102 | 1.90 |
| **L-LDH**  L-lactate dehydrogenase (EC:1.1.1.27) | Bt : XP_003396770.1 | GACGTTCAATCTCTGCGACA | CTGCCAAAGAACCGAGTGAT | 84 | 1.85 |
| **Lysozyme** | Bt: XM_003394052.2 | GTGCAGCTCGTGCAGTAGAG | TCATCCGTAGCTCCTAATTGATG | 116 | 1.85 |
| **MRJPs**  Major Royal Jelly Proteins | Bt: ADW82102.1 | TGCTGCAACAAGTTGAAATACC | CTGCATCCGCTATGTACACG | 132 | 1.92 |
| **PGRP**  Peptidoglycan  Recognition Protein S1 | Bl: JQ736605.1 | GTTGGCGAAGATGGAAACAT | ATGCAAATTCCGATGCTCTT | 95 | 2.01 |
| **PPO**  Prophenoloxidase | Bt: XM_003400500.2 | GACCGAGAAAGATGGGATCA | TGGCGACATGACCAAAGTTA | 107 | 1.93 |
| **Protein lethal (2) essential for life-like** | Bt: XM_012314610.2 | GAGGGAAAGCACGAAGAGAA | ACTTGATCGACATCGCATTG | 98 | 1.91 |
| **Relish** | Bt: XM_003399472.2 | CACAATGATGCTGGATGGAC | TGTAATGCTGTTCGACCATAAGAC | 131 | 1.83 |
| **Serpin-5** | Bt: XM_003398376.2 | CGTCCTTCTTCCACCATCTG | AGAGATCGCGGAGTTCTGTG | 134 | 1.80 |
| **Vitellogenin** | Bt: XM_012308109.1 | GAAGGCTGGGACACTGAGT | CCTGATCACGCCATGTTTC | 102 | 1.96 |
| **Actin 5C** | Bi:  XM_003488437^1^ | TTTCGCTATATGCTTCTGGACGTA | AGCGTATCCTTCGTAGATTGGTAC | 92 | 1.86 |
| **AK**  Arginine kinase | Bl: AF492871.1 | GATCCACCCTCCTTGATGTG | CAATGATGGGATCGAAGAGG | 120 | 1.97 |

Bi: *Bombus impatiens*; Bl: *B. lapidarius*; Bt: *B. terrestris*

^[[3]](#footnote-3)^ Primer sequences drawn from Simmons and Angelini (2017)

**Table S4.** Kruskal-Wallis test results describing the effect of the level of urbanization on the characteristics of the sites and local pathogens prevalence.

|  | **KW Chi-squared** | **df** | **P-value** |
| --- | --- | --- | --- |
| *B. lapidarius* abundance | 3.38 | 2 | 0.18 |
| *A. mellifera* abundance | 0.62 | 2 | 0.73 |
| Eutrophic ratio (ER) | 5.69 | 2 | 0.06 |
| Arsenic | 1.81 | 2 | 0.41 |
| Cadmium | 1.11 | 2 | 0.57 |
| Lead | 4.69 | 2 | 0.1 |
| Mercury | 3.22 | 2 | 0.2 |
| MIR | 0.22 | 2 | 0.89 |
| Apicystis prevalence | 1.69 | 2 | 0.43 |
| SBPV prevalence | 0.07 | 2 | 0.97 |
| ABPV prevalence | 2.44 | 2 | 0.29 |

**Table S5.** Spearman's rho rank correlation coefficients between pathogens prevalence, biotic and abiotic characteristics of the study sites

|  | Blap_  Apicystis | Blap_SBPV | Blap_  ABPV | imperv | BL_  abund | Am_  abund | As | Cd | Ld | Hg | MIR | ER |
| --- | --- | --- | --- | --- | --- | --- | --- | --- | --- | --- | --- | --- |
| rho | | | | | | | | | | | | |
| Blap_Apicystis | 1,00 | 0,66 | 0,20 | -0,01 | 0,15 | -0,08 | 0,40 | -0,16 | 0,24 | -0,05 | 0,00 | 0,08 |
| Blap_SBPV | 0,66 | 1,00 | 0,12 | -0,09 | 0,11 | -0,09 | 0,56 | 0,39 | 0,68 | 0,16 | 0,43 | 0,17 |
| Blap_ABPV | 0,20 | 0,12 | 1,00 | -0,52 | 0,48 | 0,17 | 0,12 | -0,36 | 0,14 | -0,19 | -0,31 | 0,23 |
| imperv | -0,01 | -0,09 | -0,52 | 1,00 | -0,59 | 0,40 | 0,19 | -0,33 | 0,38 | 0,48 | -0,12 | -0,85 |
| BL_abund | 0,15 | 0,11 | 0,48 | -0,59 | 1,00 | 0,01 | -0,36 | -0,26 | -0,48 | 0,12 | -0,22 | 0,41 |
| Am_abund | -0,08 | -0,09 | 0,17 | 0,40 | 0,01 | 1,00 | -0,43 | -0,38 | 0,00 | 0,00 | -0,55 | -0,32 |
| As | 0,40 | 0,56 | 0,12 | 0,19 | -0,36 | -0,43 | 1,00 | 0,31 | 0,81 | 0,45 | 0,67 | -0,14 |
| Cd | -0,16 | 0,39 | -0,36 | -0,33 | -0,26 | -0,38 | 0,31 | 1,00 | 0,26 | 0,10 | 0,86 | 0,55 |
| Ld | 0,24 | 0,68 | 0,14 | 0,38 | -0,48 | 0,00 | 0,81 | 0,26 | 1,00 | 0,45 | 0,45 | -0,24 |
| Hg | -0,05 | 0,16 | -0,19 | 0,48 | 0,12 | 0,00 | 0,45 | 0,10 | 0,45 | 1,00 | 0,45 | -0,26 |
| MIR | 0,00 | 0,43 | -0,31 | -0,12 | -0,22 | -0,55 | 0,67 | 0,86 | 0,45 | 0,45 | 1,00 | 0,29 |
| ER | 0,08 | 0,17 | 0,23 | -0,85 | 0,41 | -0,32 | -0,14 | 0,55 | -0,24 | -0,26 | 0,29 | 1,00 |
| n | | | | | | | | | | | | |
| Blap_Apicystis | 9 | 9 | 9 | 9 | 9 | 9 | 8 | 8 | 8 | 8 | 8 | 9 |
| Blap_SBPV | 9 | 9 | 9 | 9 | 9 | 9 | 8 | 8 | 8 | 8 | 8 | 9 |
| Blap_ABPV | 9 | 9 | 9 | 9 | 9 | 9 | 8 | 8 | 8 | 8 | 8 | 9 |
| imperv | 9 | 9 | 9 | 9 | 9 | 9 | 8 | 8 | 8 | 8 | 8 | 9 |
| BL_abund | 9 | 9 | 9 | 9 | 9 | 9 | 8 | 8 | 8 | 8 | 8 | 9 |
| Am_abund | 9 | 9 | 9 | 9 | 9 | 9 | 8 | 8 | 8 | 8 | 8 | 9 |
| As | 8 | 8 | 8 | 8 | 8 | 8 | 8 | 8 | 8 | 8 | 8 | 8 |
| Cd | 8 | 8 | 8 | 8 | 8 | 8 | 8 | 8 | 8 | 8 | 8 | 8 |
| Ld | 8 | 8 | 8 | 8 | 8 | 8 | 8 | 8 | 8 | 8 | 8 | 8 |
| Hg | 8 | 8 | 8 | 8 | 8 | 8 | 8 | 8 | 8 | 8 | 8 | 8 |
| MIR | 8 | 8 | 8 | 8 | 8 | 8 | 8 | 8 | 8 | 8 | 8 | 8 |
| ER | 9 | 9 | 9 | 9 | 9 | 9 | 8 | 8 | 8 | 8 | 8 | 9 |
| P | | | | | | | | | | | | |
| Blap_Apicystis |  | 0,051 | 0,609 | 0,983 | 0,696 | 0,847 | 0,333 | 0,713 | 0,568 | 0,910 | 1,000 | 0,830 |
| Blap_SBPV | 0,051 |  | 0,750 | 0,827 | 0,775 | 0,827 | 0,145 | 0,336 | 0,066 | 0,706 | 0,288 | 0,660 |
| Blap_ABPV | 0,609 | 0,750 |  | 0,152 | 0,192 | 0,667 | 0,779 | 0,385 | 0,736 | 0,651 | 0,456 | 0,559 |
| imperv | 0,983 | 0,827 | 0,152 |  | 0,092 | 0,286 | 0,651 | 0,420 | 0,352 | 0,233 | 0,779 | **0,004** |
| BL_abund | 0,696 | 0,775 | 0,192 | 0,092 |  | 0,983 | 0,382 | 0,528 | 0,230 | 0,778 | 0,608 | 0,273 |
| Am_abund | 0,847 | 0,827 | 0,667 | 0,286 | 0,983 |  | 0,289 | 0,352 | 1,000 | 1,000 | 0,160 | 0,406 |
| As | 0,333 | 0,145 | 0,779 | 0,651 | 0,382 | 0,289 |  | 0,456 | **0,015** | 0,260 | 0,071 | 0,736 |
| Cd | 0,713 | 0,336 | 0,385 | 0,420 | 0,528 | 0,352 |  |  | 0,531 | 0,823 | **0,007** | 0,160 |
| Ld | 0,568 | 0,066 | 0,736 | 0,352 | 0,230 | 1,000 | **0,015** | 0,531 |  | 0,260 | 0,260 | 0,570 |
| Hg | 0,910 | 0,706 | 0,651 | 0,233 | 0,778 | 1,000 | 0,260 | 0,823 | 0,260 |  | 0,260 | 0,531 |
| MIR | 1,000 | 0,288 | 0,456 | 0,779 | 0,608 | 0,160 | 0,071 | **0,007** | 0,260 | 0,260 |  | 0,493 |
| ER | 0,830 | 0,660 | 0,559 | **0,004** | 0,273 | 0,406 | 0,736 | 0,160 | 0,570 | 0,531 | 0,493 |  |

rho = matrix of correlations; n = matrix of the numbers of observations used in analyzing each pair of variables; P = matrix of asymptotic P-values (values < 0.05, in bold).

Blap_Apicystis = prevalence of *A. bombi*; Blap_SBPV = prevalence of SBPV; Blap_ABPV = prevalence of ABPV; imperv = proportion of impervious surfaces; Bl_abund = *B. lapidarius* abundance; AM_abund = *Apis mellifera* abundance; As = arsenic; Cd = cadmium; Ld = lead; Hg = mercury; MIR = Mean Impregnation Ratio; ER = Eutrophic ratio

**Table S6.** Spearman's rho rank correlation coefficients between pathogen relative loads

|  | Nb of coinf. | Apicystis | SBPV | ABPV |
| --- | --- | --- | --- | --- |
| rho | | | | |
| Nb of coinf. | 1.00 | 0.65 | 0.47 | 0.54 |
| Apicystis | 0.65 | 1.00 | -0.04 | -0.01 |
| SBPV | 0.47 | -0.04 | 1.00 | 0.41 |
| ABPV | 0.54 | -0.01 | 0.41 | 1.00 |
| n | | | | |
| Nb of coinf. | 57 | 57 | 57 | 57 |
| Apicystis | 57 | 59 | 59 | 57 |
| SBPV | 57 | 59 | 59 | 57 |
| ABPV | 57 | 57 | 57 | 57 |
| P | | | | |
| Nb of coinf. |  | **0.0000** | **0.0002** | **0.0000** |
| Apicystis | **0.0000** |  | 0.7365 | 0.9516 |
| SBPV | **0.0002** | 0.7365 |  | **0.0015** |
| ABPV | **0.0000** | 0.9516 | **0.0015** |  |

Nb of coinf. = number of coinfections

rho = matrix of correlations; n = matrix of the numbers of observations used in analyzing each pair of variables; P = matrix of asymptotic P-values (values < 0.05, in bold).

**Table S7.** Model coefficients obtained from zero-inflated GLMMs with Gamma error distribution and log-link function on pathogen loads of Apicystis, SBPV and ABPV. Urbanization levels low and medium are tested against urbanization level high.

| **Model** | **Variable** | **Estimate** | **SE** | ***z* value** | **Pr > \| *z* \|** |
| --- | --- | --- | --- | --- | --- |
| *Apicystis* |  |  |  |  |  |
|  | Intercept | −2.394 | 1.674 | −1.430 | 0.153 |
|  | Urb level low | −1.293 | 1.271 | −1.018 | 0.309 |
|  | Urb level medium | −1.215 | 1.441 | −0.843 | 0.399 |
|  | *B. lapidarius* abundance | 0.050 | 0.093 | 0.539 | 0.590 |
|  | *A. mellifera* abundance | 0.057 | 0.114 | 0.502 | 0.616 |
|  | MIR | 0.581 | 1.614 | 0.360 | 0.719 |
| *SBPV* |  |  |  |  |  |
|  | Intercept | −2.076 | 0.971 | −2.137 | **0.033** |
|  | Urb level low | 0.762 | 0.756 | 1.009 | 0.313 |
|  | Urb level medium | 0.032 | 0.702 | 0.045 | 0.964 |
|  | *B. lapidarius* abundance | −0.050 | 0.069 | −0.727 | 0.467 |
|  | *A. mellifera* abundance | 0.041 | 0.070 | 0.587 | 0.557 |
|  | MIR | −0.202 | 0.919 | −0.220 | 0.826 |
| *ABPV* |  |  |  |  |  |
|  | Intercept | −3.102 | 1.403 | −2.211 | **0.027** |
|  | Urb level low | −0.461 | 1.008 | −0.458 | 0.647 |
|  | Urb level medium | −0.669 | 1.058 | −0.632 | 0.528 |
|  | *B. lapidarius* abundance | −0.020 | 0.080 | −0.252 | 0.801 |
|  | *A. mellifera* abundance | 0.025 | 0.091 | 0.271 | 0.786 |
|  | MIR | 0.406 | 1.224 | 0.331 | 0.740 |

**Table S8.** Results of the Principal Component Analysis made on the individual pathogen loads

R package: FactoMineR

Dependent variables: Apicystis load, SBPV load, ABPV load

Quantitative illustrative variables: MIR, ER

Qualitative illustrative variables: urbanization level (low, med, high), nb of coinfections (0, 1, 2, 3)

> pca$eig

eigenvalue percentage of variance cumulative percentage of variance

comp 1 1.3052703 43.50901 43.50901

comp 2 0.9874676 32.91559 76.42460

comp 3 0.7072621 23.57540 100.00000

> summary(pca, nbelements=Inf)

Call:

PCA(X = df.cr1, quanti.sup = c(4:5), quali.sup = c(6:7))

Eigenvalues

Dim.1 Dim.2 Dim.3

Variance 1.305 0.987 0.707

% of var. 43.509 32.916 23.575

Cumulative % of var. 43.509 76.425 100.000

Individuals

Dist Dim.1 ctr cos2 Dim.2 ctr cos2 Dim.3 ctr cos2

1LBM1 | 1.042 | -0.691 0.581 0.440 | -0.722 0.837 0.480 | -0.295 0.195 0.080 |

1LBM2 | 0.729 | -0.332 0.134 0.207 | -0.567 0.516 0.604 | -0.317 0.225 0.189 |

1LBM3 | 0.891 | -0.474 0.273 0.282 | -0.553 0.492 0.385 | -0.514 0.593 0.333 |

1LBM4 | 0.847 | 0.083 0.008 0.010 | -0.216 0.075 0.065 | -0.815 1.490 0.925 |

1LBM5 | 1.042 | -0.691 0.581 0.440 | -0.722 0.837 0.480 | -0.295 0.195 0.080 |

1LBM6 | 0.779 | -0.100 0.012 0.016 | -0.333 0.179 0.183 | -0.697 1.090 0.801 |

1LBM8 | 1.246 | 0.906 0.999 0.529 | -0.178 0.051 0.020 | 0.836 1.569 0.450 |

2LBM1 | 3.086 | -1.795 3.917 0.338 | 2.420 9.413 0.615 | 0.668 1.001 0.047 |

2LBM2 | 3.361 | -1.783 3.865 0.281 | 2.730 11.979 0.660 | 0.815 1.491 0.059 |

2LBM3 | 1.403 | 1.008 1.237 0.517 | -0.448 0.322 0.102 | 0.866 1.685 0.381 |

2LBM4 | 0.787 | -0.337 0.138 0.183 | -0.500 0.402 0.404 | -0.505 0.573 0.412 |

2LBM5 | 1.211 | -0.638 0.496 0.278 | 0.921 1.363 0.579 | 0.458 0.471 0.143 |

2LBM6 | 0.502 | 0.061 0.005 0.015 | -0.491 0.388 0.959 | -0.080 0.015 0.026 |

2LBM8 | 0.000 | 0.000 0.000 0.032 | 0.000 0.000 0.956 | 0.000 0.000 0.013 |

3LBM1 | 0.360 | 0.025 0.001 0.005 | -0.350 0.197 0.948 | 0.078 0.014 0.047 |

3LBM2 | 3.098 | -1.789 3.891 0.333 | 2.336 8.772 0.569 | 0.970 2.111 0.098 |

3LBM3 | 0.617 | -0.136 0.022 0.049 | -0.461 0.342 0.560 | -0.386 0.334 0.391 |

3LBM4 | 0.362 | 0.270 0.089 0.557 | 0.180 0.052 0.248 | 0.160 0.057 0.195 |

3LBM5 | 0.763 | 0.449 0.245 0.346 | -0.495 0.395 0.422 | 0.367 0.303 0.232 |

3LBM6 | 6.817 | 5.061 31.143 0.551 | 2.829 12.869 0.172 | -3.585 28.844 0.277 |

3LBM8 | 0.000 | 0.000 0.000 0.032 | 0.000 0.000 0.956 | 0.000 0.000 0.013 |

4LBM1 | 0.811 | -0.392 0.186 0.233 | -0.710 0.811 0.767 | 0.011 0.000 0.000 |

4LBM2 | 0.822 | -0.587 0.418 0.509 | -0.406 0.264 0.243 | -0.409 0.375 0.247 |

4LBM3 | 0.884 | 0.305 0.113 0.119 | -0.123 0.024 0.019 | -0.820 1.509 0.861 |

4LBM4 | 1.316 | 0.941 1.076 0.511 | -0.452 0.329 0.118 | 0.802 1.444 0.371 |

4LBM5 | 0.616 | 0.313 0.119 0.258 | -0.491 0.387 0.636 | 0.201 0.091 0.106 |

4LBM6 | 3.809 | 3.667 16.352 0.927 | 1.017 1.661 0.071 | -0.167 0.063 0.002 |

4LBM7 | 0.000 | 0.000 0.000 0.032 | 0.000 0.000 0.956 | 0.000 0.000 0.013 |

5LBM1 | 0.351 | -0.106 0.014 0.091 | -0.315 0.159 0.807 | 0.112 0.028 0.102 |

5LBM2 | 0.795 | 0.230 0.064 0.084 | 0.720 0.834 0.820 | -0.247 0.137 0.096 |

5LBM3 | 3.306 | -0.873 0.927 0.070 | 3.178 16.234 0.924 | -0.263 0.156 0.006 |

5LBM4 | 2.873 | -0.693 0.585 0.058 | 2.728 11.962 0.901 | 0.576 0.746 0.040 |

5LBM5 | 1.042 | -0.691 0.581 0.440 | -0.722 0.837 0.480 | -0.295 0.195 0.080 |

5LBM6 | 0.707 | -0.255 0.079 0.130 | -0.490 0.385 0.479 | -0.442 0.439 0.391 |

5LBM10 | 0.000 | 0.000 0.000 0.032 | 0.000 0.000 0.956 | 0.000 0.000 0.013 |

6LBM1 | 0.908 | -0.508 0.314 0.314 | -0.580 0.541 0.408 | -0.479 0.515 0.278 |

6LBM2 | 4.762 | 3.170 12.218 0.443 | 0.040 0.003 0.000 | 3.553 28.337 0.557 |

6LBM3 | 3.081 | 2.442 7.252 0.628 | -0.224 0.080 0.005 | 1.865 7.808 0.366 |

6LBM4 | 0.792 | -0.670 0.546 0.716 | -0.260 0.109 0.108 | -0.333 0.248 0.176 |

6LBM5 | 1.120 | -0.859 0.898 0.588 | 0.716 0.825 0.409 | 0.063 0.009 0.003 |

6LBM6 | 1.098 | 0.095 0.011 0.008 | -0.111 0.020 0.010 | -1.088 2.656 0.982 |

6LBM7 | 0.891 | -0.474 0.273 0.283 | -0.553 0.492 0.385 | -0.514 0.593 0.332 |

7LBM1 | 1.042 | -0.691 0.581 0.440 | -0.722 0.837 0.480 | -0.295 0.195 0.080 |

7LBM2 | 0.904 | -0.535 0.349 0.351 | -0.716 0.824 0.627 | -0.136 0.041 0.023 |

7LBM3 | 1.042 | -0.691 0.581 0.440 | -0.722 0.837 0.480 | -0.295 0.195 0.080 |

7LBM4 | 0.759 | -0.200 0.048 0.069 | -0.703 0.794 0.857 | 0.207 0.096 0.074 |

7LBM5 | 1.545 | -1.247 1.891 0.651 | 0.840 1.134 0.296 | 0.356 0.284 0.053 |

7LBM6 | 1.021 | 0.610 0.453 0.358 | -0.524 0.442 0.264 | 0.628 0.885 0.379 |

7LBM8 | 1.675 | 0.852 0.883 0.259 | -0.662 0.706 0.156 | 1.281 3.682 0.585 |

8LBM1 | 1.042 | -0.691 0.581 0.440 | -0.722 0.837 0.480 | -0.295 0.195 0.080 |

8LBM2 | 1.042 | -0.691 0.581 0.440 | -0.722 0.837 0.480 | -0.295 0.195 0.080 |

8LBM3 | 0.547 | 0.335 0.136 0.375 | -0.294 0.139 0.288 | -0.317 0.225 0.336 |

8LBM4 | 0.567 | -0.100 0.012 0.031 | -0.490 0.385 0.746 | -0.267 0.160 0.223 |

8LBM5 | 0.660 | -0.241 0.070 0.133 | -0.533 0.457 0.653 | -0.305 0.209 0.214 |

8LBM6 | 0.494 | 0.061 0.005 0.015 | -0.472 0.357 0.910 | -0.135 0.041 0.075 |

8LBM8 | 0.695 | -0.210 0.054 0.091 | -0.458 0.337 0.435 | -0.478 0.513 0.473 |

9LBM1 | 0.714 | -0.307 0.115 0.185 | -0.550 0.486 0.593 | -0.336 0.253 0.221 |

9LBM2 | 0.456 | 0.116 0.016 0.065 | -0.342 0.188 0.562 | 0.279 0.174 0.373 |

9LBM3 | 0.694 | -0.060 0.004 0.007 | -0.354 0.202 0.260 | -0.594 0.792 0.732 |

9LBM4 | 0.540 | -0.031 0.001 0.003 | -0.452 0.328 0.700 | -0.294 0.194 0.297 |

9LBM5 | 1.845 | -1.045 1.329 0.321 | 1.426 3.270 0.598 | 0.526 0.620 0.081 |

9LBM6 | 1.830 | 1.304 2.067 0.507 | -0.449 0.324 0.060 | 1.203 3.250 0.432 |

9LBM7 | 1.042 | -0.691 0.581 0.440 | -0.722 0.837 0.480 | -0.295 0.195 0.080 |

Variables

Dim.1 ctr cos2 Dim.2 ctr cos2 Dim.3 ctr cos2

Apicystis | -0.410 12.903 0.168 | 0.872 76.936 0.760 | 0.268 10.162 0.072 |

SBPV | 0.706 38.179 0.498 | 0.476 22.992 0.227 | -0.524 38.829 0.275 |

ABPV | 0.799 48.918 0.639 | 0.027 0.072 0.001 | 0.601 51.009 0.361 |

Supplementary continuous variables

Dim.1 cos2 Dim.2 cos2 Dim.3 cos2

MIR | -0.047 0.002 | -0.075 0.006 | 0.062 0.004 |

ER | -0.117 0.014 | 0.101 0.010 | -0.125 0.016 |

Supplementary categories

Dist Dim.1 cos2 v.test Dim.2 cos2 v.test Dim.3 cos2 v.test

low | 0.281 | -0.043 0.023 -0.209 | 0.256 0.829 1.435 | -0.108 0.148 -0.716 |

med | 0.328 | 0.095 0.084 0.463 | -0.300 0.833 -1.680 | -0.095 0.083 -0.626 |

high | 0.214 | -0.052 0.059 -0.254 | 0.044 0.042 0.245 | 0.203 0.899 1.342 |

coinfections_0 | 1.042 | -0.691 0.440 -1.817 | -0.722 0.480 -2.181 | -0.295 0.080 -1.053 |

coinfections_1 | 0.628 | -0.204 0.106 -0.537 | -0.574 0.834 -1.733 | -0.154 0.060 -0.550 |

coinfections_2 | 0.277 | 0.241 0.760 1.585 | 0.079 0.082 0.599 | -0.110 0.158 -0.983 |

coinfections_3 | 0.787 | 0.026 0.001 0.083 | 0.504 0.411 1.838 | 0.603 0.588 2.597 |

> dimdesc(pca)

$Dim.1

Link between the variable and the continuous variables (R-square)

=================================================================================

correlation p.value

ABPV 0.7990723 4.195506e-15

SBPV 0.7059294 1.034260e-10

Apicystis -0.4103869 8.360302e-04

$Dim.2

Link between the variable and the continuous variables (R-square)

=================================================================================

correlation p.value

Apicystis 0.8716155 1.504030e-20

SBPV 0.4764886 7.892618e-05

Link between the variable and the categorical variable (1-way anova)

=============================================

R2 p.value

coinfections 0.173198 0.02423476

Link between variable and the categories of the categorical variables

================================================================

Estimate p.value

coinfections=coinfections_0 -0.6605793 0.02795724

$Dim.3

Link between the variable and the continuous variables (R-square)

=================================================================================

correlation p.value

ABPV 0.6006420 1.949525e-07

Apicystis 0.2680840 3.364563e-02

SBPV -0.5240442 1.043957e-05

Link between variable and the categories of the categorical variables

================================================================

Estimate p.value

coinfections=coinfections_3 0.5854847 0.008308685

**Table S9.** Spearman correlation coefficients between individual gene expression

> Spearman’s rho

AADAT abaecin apidaecin apolipoIII Defensin.1 GOX HSP90 hymenoptaecin L.LDH

AADAT 1.00 0.36 0.40 0.22 0.53 0.31 0.37 0.41 0.60

abaecin 0.36 1.00 0.57 0.62 0.88 0.10 0.41 0.76 0.44

apidaecin 0.40 0.57 1.00 0.09 0.67 0.17 0.05 0.58 0.18

apolipoIII 0.22 0.62 0.09 1.00 0.48 0.17 0.71 0.34 0.44

Defensin.1 0.53 0.88 0.67 0.48 1.00 0.18 0.37 0.80 0.46

GOX 0.31 0.10 0.17 0.17 0.18 1.00 0.28 0.07 0.09

HSP90 0.37 0.41 0.05 0.71 0.37 0.28 1.00 0.24 0.51

hymenoptaecin 0.41 0.76 0.58 0.34 0.80 0.07 0.24 1.00 0.39

L.LDH 0.60 0.44 0.18 0.44 0.46 0.09 0.51 0.39 1.00

Lysozyme 0.28 0.67 0.02 0.91 0.54 0.13 0.74 0.42 0.47

MRJP 0.56 0.39 0.34 0.47 0.52 0.37 0.54 0.26 0.60

PGRPS1 0.48 0.67 0.33 0.73 0.65 0.30 0.75 0.44 0.57

PPO 0.02 0.13 0.03 0.26 0.02 0.15 0.24 0.01 0.03

proletess 0.35 0.64 0.08 0.87 0.55 0.20 0.83 0.43 0.52

relish 0.44 0.66 0.13 0.86 0.59 0.21 0.83 0.45 0.58

serpin.5 0.64 0.55 0.43 0.56 0.63 0.29 0.73 0.50 0.55

VG 0.07 -0.05 0.20 -0.03 -0.05 -0.02 -0.12 -0.08 0.01

lysozyme MRJP PGRPS1 PPO proletess relish serpin.5 VG

AADAT 0.28 0.56 0.48 0.02 0.35 0.44 0.64 0.07

abaecin 0.67 0.39 0.67 0.13 0.64 0.66 0.55 -0.05

apidaecin 0.02 0.34 0.33 0.03 0.08 0.13 0.43 0.20

apolipoIII 0.91 0.47 0.73 0.26 0.87 0.86 0.56 -0.03

Defensin.1 0.54 0.52 0.65 0.02 0.55 0.59 0.63 -0.05

GOX 0.13 0.37 0.30 0.15 0.20 0.21 0.29 -0.02

HSP90 0.74 0.54 0.75 0.24 0.83 0.83 0.73 -0.12

hymenoptaecin 0.42 0.26 0.44 0.01 0.43 0.45 0.50 -0.08

L.LDH 0.47 0.60 0.57 0.03 0.52 0.58 0.55 0.01

Lysozyme 1.00 0.41 0.75 0.19 0.93 0.93 0.58 -0.18

MRJP 0.41 1.00 0.58 0.11 0.45 0.57 0.65 0.07

PGRPS1 0.75 0.58 1.00 0.25 0.81 0.82 0.82 0.04

PPO 0.19 0.11 0.25 1.00 0.20 0.21 0.31 -0.03

proletess 0.93 0.45 0.81 0.20 1.00 0.95 0.65 -0.16

relish 0.93 0.57 0.82 0.21 0.95 1.00 0.72 -0.20

serpin.5 0.58 0.65 0.82 0.31 0.65 0.72 1.00 0.10

VG -0.18 0.07 0.04 -0.03 -0.16 -0.20 0.10 1.00

N=63

$p

AADAT abaecin apidaecin apolipoIII Defensin.1

AADAT 0.000000000 3.178527e-01 0.168603934 9.520185e-01 3.964986e-02

abaecin 0.317852679 0.000000e+00 0.102076006 1.351795e-02 4.644208e-08

apidaecin 0.168603934 1.020760e-01 0.000000000 8.888006e-02 7.946821e-03

apolipoIII 0.952018462 1.351795e-02 0.088880062 0.000000e+00 1.532938e-01

Defensin.1 0.039649855 4.644208e-08 0.007946821 1.532938e-01 0.000000e+00

GOX 0.552811086 2.125371e-01 0.643386987 6.105158e-01 4.572894e-01

HSP90 0.427288395 1.288381e-01 0.051024471 2.326691e-06 3.390067e-01

hymenoptaecin 0.114329526 2.608845e-06 0.005451613 3.818253e-01 3.545193e-08

L.LDH 0.003191353 6.931835e-02 0.767944458 3.766686e-02 4.937225e-02

Lysozyme 0.737105147 4.256712e-03 0.143922762 1.435340e-12 6.659701e-02

MRJP 0.003859135 2.960149e-01 0.914593581 1.203288e-01 1.096304e-01

PGRPS1 0.186126476 3.117368e-03 0.565427062 4.593613e-06 1.625956e-02

PPO 0.073019315 3.592373e-01 0.077997060 5.876096e-01 1.031308e-01

proletess 0.572115268 6.783775e-03 0.147059772 9.928283e-11 7.040994e-02

relish 0.344027887 5.177851e-03 0.214977781 3.994004e-09 4.302999e-02

serpin.5 0.010558843 2.018923e-02 0.852081583 7.515834e-03 1.311866e-02

VG 0.391538662 1.809226e-02 0.581261600 5.954860e-03 5.255361e-02

GOX HSP90 hymenoptaecin L.LDH lysozyme

AADAT 0.5528111 4.272884e-01 1.143295e-01 0.0031913531 7.371051e-01

abaecin 0.2125371 1.288381e-01 2.608845e-06 0.0693183544 4.256712e-03

apidaecin 0.6433870 5.102447e-02 5.451613e-03 0.7679444581 1.439228e-01

apolipoIII 0.6105158 2.326691e-06 3.818253e-01 0.0376668589 1.435340e-12

Defensin.1 0.4572894 3.390067e-01 3.545193e-08 0.0493722503 6.659701e-02

GOX 0.0000000 7.501940e-01 2.422756e-01 0.5420147932 6.138819e-01

HSP90 0.7501940 0.000000e+00 7.469256e-01 0.0086952438 1.581636e-06

hymenoptaecin 0.2422756 7.469256e-01 0.000000e+00 0.1471813164 1.874260e-01

L.LDH 0.5420148 8.695244e-03 1.471813e-01 0.0000000000 1.687104e-02

Lysozyme 0.6138819 1.581636e-06 1.874260e-01 0.0168710383 0.000000e+00

MRJP 0.3291026 1.593759e-02 5.516924e-01 0.0008032738 1.082235e-01

PGRPS1 0.9155963 1.266969e-06 1.208850e-01 0.0029695406 8.228170e-07

PPO 0.9224291 5.257826e-01 1.213395e-01 0.2416718333 7.326669e-01

proletess 0.7620892 6.481283e-08 2.097003e-01 0.0100273136 7.338309e-15

relish 0.8235081 3.883118e-08 1.659610e-01 0.0033641027 6.306436e-12

serpin.5 0.9718596 2.017116e-04 9.008731e-02 0.0018353333 3.012519e-03

VG 0.3947911 4.320804e-03 8.657844e-02 0.0771707525 1.472689e-03

MRJP PGRPS1 PPO proletess relish

AADAT 0.0038591353 1.861265e-01 0.07301931 5.721153e-01 3.440279e-01

abaecin 0.2960149497 3.117368e-03 0.35923729 6.783775e-03 5.177851e-03

apidaecin 0.9145935811 5.654271e-01 0.07799706 1.470598e-01 2.149778e-01

apolipoIII 0.1203288327 4.593613e-06 0.58760963 9.928283e-11 3.994004e-09

Defensin.1 0.1096303757 1.625956e-02 0.10313083 7.040994e-02 4.302999e-02

GOX 0.3291026155 9.155963e-01 0.92242908 7.620892e-01 8.235081e-01

HSP90 0.0159375924 1.266969e-06 0.52578265 6.481283e-08 3.883118e-08

hymenoptaecin 0.5516924091 1.208850e-01 0.12133951 2.097003e-01 1.659610e-01

L.LDH 0.0008032738 2.969541e-03 0.24167183 1.002731e-02 3.364103e-03

Lysozyme 0.1082235284 8.228170e-07 0.73266691 7.338309e-15 6.306436e-12

MRJP 0.0000000000 1.006058e-02 0.36241216 6.791264e-02 2.331439e-02

PGRPS1 0.0100605829 0.000000e+00 0.96758077 8.028272e-08 1.290718e-08

PPO 0.3624121565 9.675808e-01 0.00000000 7.601571e-01 8.197308e-01

proletess 0.0679126370 8.028272e-08 0.76015712 0.000000e+00 1.158215e-14

relish 0.0233143865 1.290718e-08 0.81973082 1.158215e-14 0.000000e+00

serpin.5 0.0010719733 2.355860e-06 0.97203379 9.486099e-04 1.987222e-04

VG 0.1498822281 6.865389e-03 0.31242362 1.161332e-03 6.142590e-04

serpin.5 VG

AADAT 1.055884e-02 0.391538662

abaecin 2.018923e-02 0.018092261

apidaecin 8.520816e-01 0.581261600

apolipoIII 7.515834e-03 0.005954860

Defensin.1 1.311866e-02 0.052553609

GOX 9.718596e-01 0.394791100

HSP90 2.017116e-04 0.004320804

hymenoptaecin 9.008731e-02 0.086578444

L.LDH 1.835333e-03 0.077170753

Lysozyme 3.012519e-03 0.001472689

MRJP 1.071973e-03 0.149882228

PGRPS1 2.355860e-06 0.006865389

PPO 9.720338e-01 0.312423622

proletess 9.486099e-04 0.001161332

relish 1.987222e-04 0.000614259

serpin.5 0.000000e+00 0.028870541

VG 2.887054e-02 0.000000000

**Table S10.** Results of the Principal Component Analysis made on all the 17 genes

R package: FactoMineR

Dependent variables: all gene expression values

Quantitative illustrative variables: MIR, ER, Apicystis load, SBPV load, ABPV load

Qualitative illustrative variables: urbanization level (low, med, high), nb of coinfections (0, 1, 2, 3)

> pca_17genes$eig

eigenvalue percentage of variance cumulative percentage of variance

comp 1 6.390817072 37.59304160 37.59304

comp 2 2.854124778 16.78896928 54.38201

comp 3 2.068812705 12.16948650 66.55150

comp 4 1.409521749 8.29130441 74.84280

comp 5 1.013510411 5.96182595 80.80463

comp 6 0.679788925 3.99875838 84.80339

comp 7 0.590554497 3.47384998 88.27724

comp 8 0.508420642 2.99070966 91.26795

comp 9 0.336024065 1.97661215 93.24456

comp 10 0.297105339 1.74767847 94.99224

comp 11 0.223706878 1.31592281 96.30816

comp 12 0.206043979 1.21202341 97.52018

comp 13 0.151866207 0.89333063 98.41351

comp 14 0.143227508 0.84251475 99.25603

comp 15 0.069405697 0.40826881 99.66430

comp 16 0.047388817 0.27875775 99.94305

comp 17 0.009680729 0.05694547 100.00000

> summary(pca_17genes, nbelements=Inf

Call:

PCA(X = df.cr, quanti.sup = c(18:22), quali.sup = 23:24)

Eigenvalues

Dim.1 Dim.2 Dim.3 Dim.4 Dim.5 Dim.6 Dim.7 Dim.8

Variance 6.391 2.854 2.069 1.410 1.014 0.680 0.591 0.508

% of var. 37.593 16.789 12.169 8.291 5.962 3.999 3.474 2.991

Cumulative % of var. 37.593 54.382 66.551 74.843 80.805 84.803 88.277 91.268

Dim.9 Dim.10 Dim.11 Dim.12 Dim.13 Dim.14 Dim.15 Dim.16

Variance 0.336 0.297 0.224 0.206 0.152 0.143 0.069 0.047

% of var. 1.977 1.748 1.316 1.212 0.893 0.843 0.408 0.279

Cumulative % of var. 93.245 94.992 96.308 97.520 98.414 99.256 99.664 99.943

Dim.17

Variance 0.010

% of var. 0.057

Cumulative % of var. 100.000

Individuals

Dist Dim.1 ctr cos2 Dim.2 ctr cos2 Dim.3 ctr

1LBM1 | 2.860 | -2.627 1.715 0.844 | -0.770 0.330 0.072 | -0.544 0.227

1LBM2 | 2.776 | 0.387 0.037 0.019 | -0.160 0.014 0.003 | 0.322 0.079

1LBM3 | 2.509 | -2.116 1.112 0.712 | 0.429 0.102 0.029 | -0.338 0.088

1LBM4 | 2.493 | -0.998 0.247 0.160 | -1.097 0.670 0.194 | 0.337 0.087

1LBM5 | 2.308 | -2.121 1.117 0.844 | -0.075 0.003 0.001 | 0.283 0.062

1LBM6 | 6.325 | 4.628 5.319 0.535 | 0.190 0.020 0.001 | -2.761 5.848

1LBM8 | 4.852 | 0.908 0.205 0.035 | -2.536 3.577 0.273 | -2.471 4.683

2LBM1 | 4.923 | 2.842 2.006 0.333 | -1.453 1.174 0.087 | 1.183 1.075

2LBM2 | 2.292 | -2.058 1.052 0.807 | -0.019 0.000 0.000 | -0.374 0.107

2LBM3 | 2.015 | -1.717 0.732 0.726 | -0.507 0.143 0.063 | 0.204 0.032

2LBM4 | 4.498 | 1.914 0.910 0.181 | 0.867 0.418 0.037 | -2.205 3.731

2LBM5 | 2.964 | -2.278 1.289 0.591 | 0.263 0.039 0.008 | -1.381 1.463

2LBM6 | 1.755 | -1.015 0.256 0.334 | 0.181 0.018 0.011 | -0.027 0.001

2LBM8 | 2.329 | -1.328 0.438 0.325 | -1.029 0.589 0.195 | -0.554 0.235

3LBM1 | 1.964 | -1.473 0.539 0.562 | 0.032 0.001 0.000 | -0.336 0.087

3LBM2 | 2.862 | -2.520 1.577 0.775 | -0.063 0.002 0.000 | -0.475 0.173

3LBM3 | 1.744 | -0.953 0.226 0.299 | -0.466 0.121 0.071 | -0.071 0.004

3LBM4 | 4.164 | 1.972 0.966 0.224 | 1.936 2.085 0.216 | 1.814 2.524

3LBM5 | 2.899 | -2.142 1.139 0.546 | 1.074 0.641 0.137 | -1.125 0.971

3LBM6 | 2.339 | -1.911 0.907 0.668 | -0.836 0.389 0.128 | -0.061 0.003

3LBM8 | 2.182 | -0.066 0.001 0.001 | -1.106 0.680 0.257 | -0.302 0.070

4LBM1 | 2.420 | -2.245 1.252 0.861 | -0.449 0.112 0.034 | 0.247 0.047

4LBM2 | 1.864 | -0.487 0.059 0.068 | -0.855 0.407 0.210 | -0.417 0.134

4LBM3 | 5.495 | 2.323 1.340 0.179 | 4.193 9.777 0.582 | -1.102 0.931

4LBM4 | 2.674 | -0.499 0.062 0.035 | -0.605 0.204 0.051 | -0.220 0.037

4LBM5 | 10.674 | 2.108 1.104 0.039 | 9.608 51.345 0.810 | -3.154 7.630

4LBM6 | 2.597 | -2.240 1.247 0.744 | -0.773 0.332 0.089 | -0.461 0.163

4LBM7 | 2.086 | -1.330 0.440 0.407 | -0.439 0.107 0.044 | -0.121 0.011

5LBM1 | 5.095 | 1.094 0.297 0.046 | 0.473 0.125 0.009 | 3.651 10.230

5LBM2 | 2.861 | -0.270 0.018 0.009 | 1.340 0.999 0.219 | -0.532 0.217

5LBM3 | 10.992 | 9.636 23.063 0.768 | -3.038 5.133 0.076 | -1.024 0.805

5LBM4 | 6.402 | 3.408 2.885 0.283 | 1.528 1.298 0.057 | 3.994 12.239

5LBM5 | 3.793 | -1.415 0.497 0.139 | -0.445 0.110 0.014 | 1.292 1.281

5LBM6 | 2.315 | -1.295 0.416 0.313 | -0.305 0.052 0.017 | 0.967 0.718

5LBM10 | 1.875 | -1.098 0.299 0.343 | 0.306 0.052 0.027 | 0.612 0.287

6LBM1 | 2.125 | -1.141 0.324 0.289 | 0.200 0.022 0.009 | 1.406 1.518

6LBM2 | 1.512 | 0.283 0.020 0.035 | -0.472 0.124 0.098 | 0.042 0.001

6LBM3 | 2.138 | -1.344 0.449 0.395 | -0.343 0.065 0.026 | -0.071 0.004

6LBM4 | 8.499 | 0.812 0.164 0.009 | 0.477 0.127 0.003 | 4.735 17.202

6LBM5 | 2.682 | 0.935 0.217 0.122 | 0.977 0.531 0.133 | 1.173 1.056

6LBM6 | 2.060 | -1.608 0.642 0.609 | -0.809 0.364 0.154 | -0.310 0.074

6LBM7 | 2.684 | 0.239 0.014 0.008 | -1.781 1.764 0.440 | -0.714 0.391

7LBM1 | 3.274 | -2.920 2.118 0.796 | -0.834 0.386 0.065 | -0.887 0.603

7LBM2 | 2.114 | -1.642 0.669 0.603 | 0.477 0.126 0.051 | 0.229 0.040

7LBM3 | 4.042 | 1.869 0.867 0.214 | 2.663 3.944 0.434 | 0.524 0.211

7LBM4 | 5.517 | 3.531 3.097 0.410 | 0.488 0.132 0.008 | 2.469 4.677

7LBM5 | 2.877 | 0.408 0.041 0.020 | 1.695 1.598 0.347 | 0.243 0.045

7LBM6 | 2.044 | -0.413 0.042 0.041 | 0.594 0.196 0.084 | 0.492 0.186

7LBM8 | 2.259 | -2.033 1.026 0.809 | -0.756 0.318 0.112 | -0.211 0.034

8LBM1 | 8.461 | 5.173 6.646 0.374 | -1.544 1.326 0.033 | -3.451 9.139

8LBM2 | 1.649 | -0.838 0.174 0.258 | -0.120 0.008 0.005 | 0.173 0.023

8LBM3 | 1.699 | -0.921 0.211 0.294 | -0.194 0.021 0.013 | -0.033 0.001

8LBM4 | 10.808 | 8.661 18.631 0.642 | -3.018 5.064 0.078 | -0.753 0.435

8LBM5 | 1.424 | -0.158 0.006 0.012 | -0.917 0.468 0.415 | -0.714 0.391

8LBM6 | 1.883 | -0.501 0.062 0.071 | -0.128 0.009 0.005 | -0.737 0.417

8LBM8 | 2.533 | -1.867 0.866 0.543 | 0.568 0.179 0.050 | -0.138 0.015

9LBM1 | 2.886 | 1.322 0.434 0.210 | 0.927 0.478 0.103 | 0.145 0.016

9LBM2 | 2.912 | -2.640 1.731 0.822 | -0.762 0.323 0.068 | -0.714 0.392

9LBM3 | 2.795 | 1.867 0.866 0.446 | -0.589 0.193 0.044 | 0.047 0.002

9LBM4 | 2.956 | -2.544 1.607 0.740 | -1.011 0.568 0.117 | -0.653 0.327

9LBM5 | 5.356 | 3.445 2.948 0.414 | 0.240 0.032 0.002 | 2.893 6.420

9LBM6 | 2.712 | -2.196 1.197 0.656 | -0.677 0.255 0.062 | -0.273 0.057

9LBM7 | 1.872 | -0.796 0.157 0.181 | -0.748 0.311 0.160 | 0.233 0.042

cos2

1LBM1 0.036 |

1LBM2 0.013 |

1LBM3 0.018 |

1LBM4 0.018 |

1LBM5 0.015 |

1LBM6 0.191 |

1LBM8 0.259 |

2LBM1 0.058 |

2LBM2 0.027 |

2LBM3 0.010 |

2LBM4 0.240 |

2LBM5 0.217 |

2LBM6 0.000 |

2LBM8 0.056 |

3LBM1 0.029 |

3LBM2 0.027 |

3LBM3 0.002 |

3LBM4 0.190 |

3LBM5 0.151 |

3LBM6 0.001 |

3LBM8 0.019 |

4LBM1 0.010 |

4LBM2 0.050 |

4LBM3 0.040 |

4LBM4 0.007 |

4LBM5 0.087 |

4LBM6 0.032 |

4LBM7 0.003 |

5LBM1 0.514 |

5LBM2 0.035 |

5LBM3 0.009 |

5LBM4 0.389 |

5LBM5 0.116 |

5LBM6 0.175 |

5LBM10 0.107 |

6LBM1 0.438 |

6LBM2 0.001 |

6LBM3 0.001 |

6LBM4 0.310 |

6LBM5 0.191 |

6LBM6 0.023 |

6LBM7 0.071 |

7LBM1 0.073 |

7LBM2 0.012 |

7LBM3 0.017 |

7LBM4 0.200 |

7LBM5 0.007 |

7LBM6 0.058 |

7LBM8 0.009 |

8LBM1 0.166 |

8LBM2 0.011 |

8LBM3 0.000 |

8LBM4 0.005 |

8LBM5 0.251 |

8LBM6 0.153 |

8LBM8 0.003 |

9LBM1 0.003 |

9LBM2 0.060 |

9LBM3 0.000 |

9LBM4 0.049 |

9LBM5 0.292 |

9LBM6 0.010 |

9LBM7 0.015 |

Variables

Dim.1 ctr cos2 Dim.2 ctr cos2 Dim.3 ctr cos2

AADAT | 0.486 3.700 0.236 | 0.261 2.380 0.068 | 0.618 18.451 0.382 |

abaecin | 0.572 5.123 0.327 | 0.386 5.217 0.149 | -0.485 11.372 0.235 |

apidaecin | 0.250 0.978 0.062 | 0.823 23.750 0.678 | -0.102 0.507 0.010 |

apolipoIII | 0.767 9.206 0.588 | -0.327 3.744 0.107 | -0.323 5.056 0.105 |

Defensin.1 | 0.516 4.164 0.266 | 0.750 19.713 0.563 | -0.316 4.814 0.100 |

GOX | 0.390 2.375 0.152 | -0.099 0.340 0.010 | 0.314 4.759 0.098 |

HSP90 | 0.861 11.610 0.742 | -0.203 1.449 0.041 | 0.069 0.229 0.005 |

hymenoptaecin | 0.172 0.462 0.030 | 0.840 24.724 0.706 | -0.295 4.202 0.087 |

L.LDH | 0.434 2.944 0.188 | 0.274 2.634 0.075 | 0.599 17.338 0.359 |

Lysozyme | 0.710 7.885 0.504 | -0.326 3.722 0.106 | -0.355 6.102 0.126 |

MRJP | 0.656 6.728 0.430 | 0.109 0.420 0.012 | 0.437 9.220 0.191 |

PGRPS1 | 0.711 7.903 0.505 | -0.152 0.807 0.023 | 0.002 0.000 0.000 |

PPO | 0.602 5.670 0.362 | -0.256 2.293 0.065 | 0.068 0.222 0.005 |

proletess | 0.810 10.267 0.656 | -0.315 3.485 0.099 | -0.222 2.385 0.049 |

relish | 0.873 11.923 0.762 | -0.293 3.002 0.086 | -0.161 1.249 0.026 |

serpin.5 | 0.761 9.064 0.579 | 0.254 2.252 0.064 | 0.349 5.881 0.122 |

VG | 0.004 0.000 0.000 | 0.044 0.069 0.002 | 0.412 8.212 0.170 |

Supplementary continuous variables

Dim.1 cos2 Dim.2 cos2 Dim.3 cos2

Apicystis | 0.273 0.074 | -0.048 0.002 | 0.213 0.046 |

SBPV | -0.006 0.000 | -0.077 0.006 | -0.051 0.003 |

ABPV | -0.166 0.028 | -0.035 0.001 | -0.062 0.004 |

MIR | -0.044 0.002 | -0.087 0.008 | 0.038 0.001 |

ER | -0.062 0.004 | -0.216 0.047 | -0.264 0.070 |

Supplementary categories

Dist Dim.1 cos2 v.test Dim.2 cos2 v.test Dim.3 cos2

low | 0.861 | -0.603 0.491 -1.329 | -0.245 0.081 -0.807 | -0.423 0.241

med | 0.611 | 0.268 0.193 0.591 | 0.129 0.045 0.425 | -0.438 0.515

high | 1.018 | 0.335 0.108 0.738 | 0.116 0.013 0.382 | 0.861 0.715

coinfections_0 | 0.991 | -0.460 0.215 -0.546 | -0.234 0.056 -0.416 | -0.297 0.090

coinfections_1 | 1.208 | -0.877 0.527 -1.042 | -0.275 0.052 -0.489 | 0.347 0.083

coinfections_2 | 0.696 | 0.404 0.337 1.199 | 0.118 0.029 0.524 | -0.259 0.138

coinfections_3 | 0.639 | -0.007 0.000 -0.011 | 0.134 0.044 0.287 | 0.342 0.288

v.test

low -1.637 |

med -1.697 |

high 3.333 |

coinfections_0 -0.620 |

coinfections_1 0.725 |

coinfections_2 -1.351 |

coinfections_3 0.862 |

> dimdesc(pca_17genes)

$Dim.1

Link between the variable and the continuous variables (R-square)

=================================================================================

correlation p.value

relish 0.8728949 1.129875e-20

HSP90 0.8613729 1.337274e-19

proletess 0.8100474 8.988103e-16

apolipoIII 0.7670175 2.302476e-13

serpin.5 0.7610909 4.504261e-13

PGRPS1 0.7106766 6.809659e-11

Lysozyme 0.7098514 7.327162e-11

MRJP 0.6557234 5.455885e-09

PPO 0.6019646 1.803205e-07

abaecin 0.5721660 9.635633e-07

Defensin.1 0.5158656 1.510936e-05

AADAT 0.4862658 5.334334e-05

L.LDH 0.4337469 3.833137e-04

GOX 0.3895548 1.600859e-03

Apicystis 0.2728900 3.047385e-02

apidaecin 0.2499862 4.815858e-02

$Dim.2

Link between the variable and the continuous variables (R-square)

=================================================================================

correlation p.value

hymenoptaecin 0.8400398 7.585032e-18

apidaecin 0.8233202 1.214029e-16

Defensin.1 0.7500900 1.489071e-12

abaecin 0.3858668 1.788277e-03

L.LDH 0.2741763 2.966876e-02

AADAT 0.2606303 3.910626e-02

serpin.5 0.2535091 4.498958e-02

PPO -0.2558113 4.301280e-02

relish -0.2927154 1.990805e-02

proletess -0.3153734 1.181301e-02

lysozyme -0.3259193 9.142006e-03

apolipoIII -0.3269015 8.922372e-03

$Dim.3

Link between the variable and the continuous variables (R-square)

=================================================================================

correlation p.value

AADAT 0.6178377 6.873082e-08

L.LDH 0.5989120 2.157818e-07

MRJP 0.4367522 3.452968e-04

VG 0.4121798 7.890108e-04

serpin.5 0.3488058 5.083484e-03

GOX 0.3137858 1.226855e-02

ER -0.2636905 3.678154e-02

hymenoptaecin -0.2948495 1.898408e-02

Defensin.1 -0.3155780 1.175536e-02

apolipoIII -0.3234146 9.723515e-03

lysozyme -0.3553082 4.268872e-03

abaecin -0.4850361 5.607427e-05

Link between the variable and the categorical variable (1-way anova)

=============================================

R2 p.value

urb_level 0.179226 0.002671209

Link between variable and the categories of the categorical variables

================================================================

Estimate p.value

urb_level=high 0.8610984 0.0005465272

**Table S11.** Results of the Principal Component Analysis made on 5 selected genes: AADAT, L-LDH, abaecin, MRJP and vg

R package: FactoMineR

Dependent variables: gene expression of AADAT, L-LDH, abaecin, MRJP, and vg genes

Quantitative illustrative variables: MIR, ER, Apicystis load, SBPV load, ABPV load

Qualitative illustrative variables: urbanization level (low, med, high), nb of coinfections (0, 1, 2, 3)

> pca_5genes$eig

eigenvalue percentage of variance cumulative percentage of variance

comp 1 2.3013193 46.026386 46.02639

comp 2 1.1142550 22.285099 68.31149

comp 3 0.8218358 16.436715 84.74820

comp 4 0.5026322 10.052644 94.80084

comp 5 0.2599578 5.199155 100.00000

> summary(pca_5genes, nbelements=Inf)

Call:

PCA(X = df.cr_res, quanti.sup = c(6:10), quali.sup = 11:12)

Eigenvalues

Dim.1 Dim.2 Dim.3 Dim.4 Dim.5

Variance 2.301 1.114 0.822 0.503 0.260

% of var. 46.026 22.285 16.437 10.053 5.199

Cumulative % of var. 46.026 68.311 84.748 94.801 100.000

Individuals

Dist Dim.1 ctr cos2 Dim.2 ctr cos2 Dim.3 ctr

1LBM1 | 1.708 | -1.649 1.875 0.932 | 0.308 0.136 0.033 | -0.122 0.029

1LBM2 | 0.983 | -0.865 0.515 0.774 | 0.151 0.033 0.024 | -0.202 0.079

1LBM3 | 0.987 | -0.938 0.607 0.904 | 0.166 0.039 0.028 | -0.244 0.115

1LBM4 | 1.156 | -1.077 0.800 0.868 | 0.187 0.050 0.026 | -0.281 0.153

1LBM5 | 0.886 | -0.444 0.136 0.252 | 0.694 0.686 0.614 | -0.128 0.032

1LBM6 | 4.153 | 1.220 1.026 0.086 | -2.878 11.797 0.480 | 2.624 13.295

1LBM8 | 1.433 | -1.326 1.213 0.856 | 0.223 0.071 0.024 | 0.118 0.027

2LBM1 | 0.891 | 0.425 0.125 0.228 | 0.217 0.067 0.059 | -0.591 0.675

2LBM2 | 1.087 | -1.052 0.764 0.938 | 0.183 0.048 0.028 | -0.192 0.071

2LBM3 | 1.093 | -0.802 0.444 0.539 | 0.265 0.100 0.059 | -0.222 0.095

2LBM4 | 3.434 | -0.318 0.070 0.009 | -2.100 6.281 0.374 | 2.506 12.126

2LBM5 | 1.831 | -1.776 2.176 0.942 | 0.106 0.016 0.003 | 0.094 0.017

2LBM6 | 0.941 | -0.912 0.573 0.939 | 0.093 0.012 0.010 | -0.123 0.029

2LBM8 | 1.215 | -1.147 0.907 0.891 | 0.119 0.020 0.010 | -0.219 0.093

3LBM1 | 1.080 | -0.721 0.359 0.446 | 0.606 0.523 0.315 | 0.509 0.500

3LBM2 | 1.583 | -1.324 1.210 0.700 | 0.634 0.572 0.160 | 0.242 0.113

3LBM3 | 1.055 | -0.940 0.609 0.793 | 0.209 0.062 0.039 | -0.281 0.153

3LBM4 | 2.632 | 2.486 4.264 0.892 | -0.382 0.208 0.021 | -0.729 1.028

3LBM5 | 1.599 | -1.569 1.699 0.964 | -0.048 0.003 0.001 | 0.201 0.078

3LBM6 | 1.415 | -1.334 1.228 0.889 | 0.333 0.158 0.055 | -0.164 0.052

3LBM8 | 1.218 | 0.082 0.005 0.005 | 0.119 0.020 0.009 | -0.149 0.043

4LBM1 | 0.868 | -0.646 0.288 0.554 | 0.271 0.105 0.097 | -0.447 0.386

4LBM2 | 0.652 | -0.522 0.188 0.640 | 0.180 0.046 0.076 | -0.335 0.216

4LBM3 | 1.712 | 1.007 0.699 0.346 | -0.944 1.269 0.304 | 0.692 0.926

4LBM4 | 2.244 | 0.149 0.015 0.004 | 0.317 0.144 0.020 | 0.314 0.191

4LBM5 | 2.669 | 0.693 0.331 0.067 | -1.684 4.041 0.398 | 1.746 5.890

4LBM6 | 1.422 | -1.354 1.265 0.907 | 0.318 0.144 0.050 | -0.138 0.037

4LBM7 | 0.935 | -0.540 0.201 0.334 | 0.213 0.065 0.052 | -0.028 0.002

5LBM1 | 3.826 | 3.268 7.368 0.730 | 0.168 0.040 0.002 | -1.733 5.801

5LBM2 | 2.221 | 0.972 0.651 0.191 | -0.547 0.426 0.061 | 1.807 6.307

5LBM3 | 2.798 | 1.633 1.838 0.340 | -1.037 1.533 0.137 | -0.476 0.438

5LBM4 | 4.891 | 4.023 11.162 0.676 | 0.085 0.010 0.000 | -1.798 6.245

5LBM5 | 1.411 | 0.038 0.001 0.001 | -0.039 0.002 0.001 | -0.675 0.880

5LBM6 | 1.312 | 0.350 0.085 0.071 | 0.907 1.172 0.478 | 0.041 0.003

5LBM10 | 0.790 | -0.002 0.000 0.000 | 0.169 0.041 0.046 | -0.285 0.157

6LBM1 | 1.391 | 0.871 0.523 0.392 | 0.372 0.197 0.072 | -0.825 1.315

6LBM2 | 0.479 | -0.223 0.034 0.216 | 0.126 0.023 0.069 | -0.292 0.165

6LBM3 | 1.168 | -0.874 0.526 0.560 | 0.261 0.097 0.050 | -0.213 0.087

6LBM4 | 8.297 | 4.074 11.450 0.241 | 6.273 56.053 0.572 | 3.512 23.824

6LBM5 | 1.665 | 1.038 0.742 0.388 | -0.445 0.282 0.071 | -0.631 0.769

6LBM6 | 1.351 | -1.142 0.900 0.714 | 0.376 0.201 0.077 | -0.145 0.041

6LBM7 | 1.073 | -0.814 0.457 0.576 | 0.319 0.145 0.089 | -0.177 0.060

7LBM1 | 1.993 | -1.930 2.570 0.938 | 0.248 0.087 0.015 | -0.100 0.019

7LBM2 | 0.667 | -0.466 0.150 0.488 | 0.235 0.079 0.124 | -0.330 0.210

7LBM3 | 2.914 | 2.567 4.545 0.776 | -1.113 1.766 0.146 | 0.316 0.192

7LBM4 | 4.868 | 4.524 14.118 0.864 | -0.098 0.014 0.000 | -1.359 3.567

7LBM5 | 0.810 | -0.084 0.005 0.011 | -0.042 0.003 0.003 | -0.021 0.001

7LBM6 | 1.517 | 1.042 0.749 0.472 | -0.161 0.037 0.011 | -0.216 0.090

7LBM8 | 1.273 | -1.185 0.969 0.867 | 0.277 0.109 0.047 | -0.201 0.078

8LBM1 | 3.516 | 1.410 1.371 0.161 | -2.243 7.169 0.407 | 1.986 7.616

8LBM2 | 0.701 | -0.245 0.041 0.122 | 0.265 0.100 0.143 | -0.300 0.174

8LBM3 | 0.829 | -0.453 0.142 0.299 | -0.071 0.007 0.007 | -0.332 0.213

8LBM4 | 1.188 | 0.447 0.138 0.141 | -0.772 0.849 0.422 | 0.406 0.318

8LBM5 | 0.799 | -0.731 0.369 0.837 | -0.145 0.030 0.033 | 0.015 0.000

8LBM6 | 1.322 | -1.148 0.909 0.754 | -0.268 0.103 0.041 | 0.162 0.051

8LBM8 | 1.358 | -0.765 0.404 0.317 | 0.219 0.068 0.026 | -0.482 0.449

9LBM1 | 2.151 | 1.199 0.991 0.310 | -0.828 0.976 0.148 | -0.022 0.001

9LBM2 | 1.781 | -1.718 2.036 0.931 | 0.350 0.174 0.039 | -0.032 0.002

9LBM3 | 1.637 | 0.462 0.147 0.080 | -0.665 0.630 0.165 | 0.059 0.007

9LBM4 | 1.924 | -1.883 2.446 0.958 | 0.317 0.143 0.027 | -0.046 0.004

9LBM5 | 4.224 | 3.065 6.481 0.527 | -0.635 0.574 0.023 | -1.406 3.817

9LBM6 | 1.786 | -1.685 1.959 0.890 | 0.313 0.140 0.031 | -0.083 0.013

9LBM7 | 1.330 | -0.438 0.133 0.109 | -0.049 0.003 0.001 | -0.574 0.637

cos2

1LBM1 0.005 |

1LBM2 0.042 |

1LBM3 0.061 |

1LBM4 0.059 |

1LBM5 0.021 |

1LBM6 0.399 |

1LBM8 0.007 |

2LBM1 0.440 |

2LBM2 0.031 |

2LBM3 0.041 |

2LBM4 0.532 |

2LBM5 0.003 |

2LBM6 0.017 |

2LBM8 0.033 |

3LBM1 0.222 |

3LBM2 0.023 |

3LBM3 0.071 |

3LBM4 0.077 |

3LBM5 0.016 |

3LBM6 0.013 |

3LBM8 0.015 |

4LBM1 0.265 |

4LBM2 0.263 |

4LBM3 0.164 |

4LBM4 0.020 |

4LBM5 0.428 |

4LBM6 0.009 |

4LBM7 0.001 |

5LBM1 0.205 |

5LBM2 0.662 |

5LBM3 0.029 |

5LBM4 0.135 |

5LBM5 0.229 |

5LBM6 0.001 |

5LBM10 0.131 |

6LBM1 0.352 |

6LBM2 0.372 |

6LBM3 0.033 |

6LBM4 0.179 |

6LBM5 0.144 |

6LBM6 0.011 |

6LBM7 0.027 |

7LBM1 0.003 |

7LBM2 0.244 |

7LBM3 0.012 |

7LBM4 0.078 |

7LBM5 0.001 |

7LBM6 0.020 |

7LBM8 0.025 |

8LBM1 0.319 |

8LBM2 0.183 |

8LBM3 0.161 |

8LBM4 0.117 |

8LBM5 0.000 |

8LBM6 0.015 |

8LBM8 0.126 |

9LBM1 0.000 |

9LBM2 0.000 |

9LBM3 0.001 |

9LBM4 0.001 |

9LBM5 0.111 |

9LBM6 0.002 |

9LBM7 0.186 |

Variables

Dim.1 ctr cos2 Dim.2 ctr cos2 Dim.3 ctr cos2

AADAT | 0.842 30.772 0.708 | -0.026 0.060 0.001 | -0.290 10.217 0.084 |

abaecin | 0.326 4.607 0.106 | -0.670 40.245 0.448 | 0.660 52.978 0.435 |

L.LDH | 0.888 34.259 0.788 | 0.174 2.721 0.030 | -0.019 0.046 0.000 |

MRJP | 0.770 25.737 0.592 | -0.215 4.133 0.046 | -0.162 3.213 0.026 |

VG | 0.326 4.625 0.106 | 0.767 52.841 0.589 | 0.525 33.546 0.276 |

Supplementary continuous variables

Dim.1 cos2 Dim.2 cos2 Dim.3 cos2

Apicystis | 0.212 0.045 | 0.010 0.000 | -0.206 0.043 |

SBPV | -0.100 0.010 | -0.013 0.000 | -0.005 0.000 |

ABPV | -0.171 0.029 | 0.052 0.003 | -0.087 0.008 |

MIR | -0.065 0.004 | -0.031 0.001 | -0.065 0.004 |

ER | -0.332 0.110 | -0.148 0.022 | 0.083 0.007 |

Supplementary categories

Dist Dim.1 cos2 v.test Dim.2 cos2 v.test Dim.3 cos2

low | 0.680 | -0.666 0.960 -2.444 | -0.038 0.003 -0.199 | 0.126 0.034

med | 0.324 | -0.176 0.296 -0.646 | -0.264 0.664 -1.392 | 0.055 0.029

high | 0.915 | 0.842 0.847 3.090 | 0.302 0.109 1.591 | -0.181 0.039

coinfections_0 | 0.300 | -0.086 0.083 -0.171 | -0.241 0.647 -0.686 | 0.050 0.028

coinfections_1 | 0.649 | 0.025 0.002 0.050 | 0.240 0.137 0.682 | -0.466 0.516

coinfections_2 | 0.351 | -0.183 0.271 -0.905 | -0.013 0.001 -0.089 | 0.288 0.671

coinfections_3 | 0.444 | 0.365 0.675 0.871 | -0.022 0.003 -0.077 | -0.226 0.260

v.test

low 0.774 |

med 0.338 |

high -1.112 |

coinfections_0 0.166 |

coinfections_1 -1.543 |

coinfections_2 2.381 |

coinfections_3 -0.904 |

> dimdesc(pca_5genes)

$Dim.1

Link between the variable and the continuous variables (R-square)

=================================================================================

correlation p.value

L.LDH 0.8879219 3.056233e-22

AADAT 0.8415248 5.839414e-18

MRJP 0.7696061 1.706869e-13

VG 0.3262479 9.068008e-03

abaecin 0.3256053 9.213211e-03

ER -0.3322624 7.802785e-03

Link between the variable and the categorical variable (1-way anova)

=============================================

R2 p.value

urb_level 0.1713663 0.003555315

Link between variable and the categories of the categorical variables

================================================================

Estimate p.value

urb_level=high 0.8419058 0.001467273

urb_level=low -0.6657973 0.013309570

$Dim.2

Link between the variable and the continuous variables (R-square)

=================================================================================

correlation p.value

VG 0.7673226 2.223099e-13

abaecin -0.6696540 1.959727e-09

$Dim.3

Link between the variable and the continuous variables (R-square)

=================================================================================

correlation p.value

abaecin 0.6598450 4.052501e-09

VG 0.5250627 9.963114e-06

AADAT -0.2897731 2.124450e-02

Link between variable and the categories of the categorical variables

================================================================

Estimate p.value

coinfections=coinfections_2 0.4521179 0.01600606

**Table S12** PLS analysis on gene expression dataset a dataset that gathers all environmental and infection data - tuning steps and supplementary results

R package: mixOmics

X = relative gene expression (17 genes)

Y = local rate of impervious surface, MIR, ER, loads of Apicystis, SBPV and ABPV

Model to be tuned: pls.result <- pls(X, Y, mode = "canonical", ncomp = 6)

> pls.result$loadings

$X

comp1 comp2 comp3 comp4 comp5 comp6

AADAT -0.491247366 0.007739185 -0.00325487 0.049177550 -0.399693805 0.17802255

abaecin -0.005987954 -0.218175073 0.46314946 0.192279284 0.392557676 -0.02641507

apidaecin -0.168379852 -0.071583049 0.38190976 0.179883209 -0.291816322 -0.67380053

apolipoIII 0.041265363 -0.107001306 0.12809506 -0.236141882 0.004797039 -0.31040903

Defensin-1 -0.182918106 -0.259781873 0.07691241 0.399493046 0.109326252 -0.08749839

GOX -0.143947770 -0.271066556 0.28142822 -0.389823840 -0.210707675 0.31710688

HSP90 -0.186447618 0.265055776 0.07643293 0.005689451 0.316603445 0.05719838

hymenoptaecin -0.088456475 -0.216866483 -0.11452285 0.312491166 -0.019156092 0.08078386

L-LDH -0.421359011 -0.403834188 -0.54319832 0.093853887 0.275982167 -0.08876053

lysozyme-3 -0.038042348 -0.011588951 0.24027499 0.092728091 -0.030661478 0.20606513

MRJP -0.382139960 0.012627743 0.08323164 -0.356808411 0.087322006 -0.09384410

PGRPS1 -0.102168120 -0.106291850 0.36683676 0.212822909 0.072678759 0.33650308

PPO -0.113283867 0.559547466 0.05934536 0.185305233 0.356506313 -0.03738647

proletess -0.255285024 0.066952584 0.02195015 -0.375969428 0.227962616 -0.27713565

relish -0.243062335 0.120737217 0.09333895 -0.185886061 0.096260996 0.19024858

serpin-5 -0.358680871 0.402670372 -0.05592759 0.246919855 -0.338022502 0.01784214

VG -0.174995587 -0.092446215 0.09490412 -0.044965575 0.226729332 0.11018972

$Y

comp1 comp2 comp3 comp4 comp5 comp6

imperv -0.5567108 -0.29504265 -0.1688501 -0.28035013 -0.07565919 0.70013720

ER 0.4311898 0.36185056 0.4621329 -0.50038865 -0.27848837 0.37633521

MIR 0.1962257 0.50459565 -0.2822913 0.44369446 0.39822746 0.52128730

Apicystis -0.5393134 0.71458754 -0.1641679 -0.10714440 -0.31675130 -0.24442442

SBPV 0.1686538 0.05962146 -0.5018250 -0.67600480 0.47582212 -0.18106258

ABPV 0.3825454 -0.11492241 -0.6320246 0.07541761 -0.65680798 0.06254842

Selection of the number of components:

perf.pls.result <- perf(pls.result, validation = 'Mfold', folds = 10, nrepeat = 100)


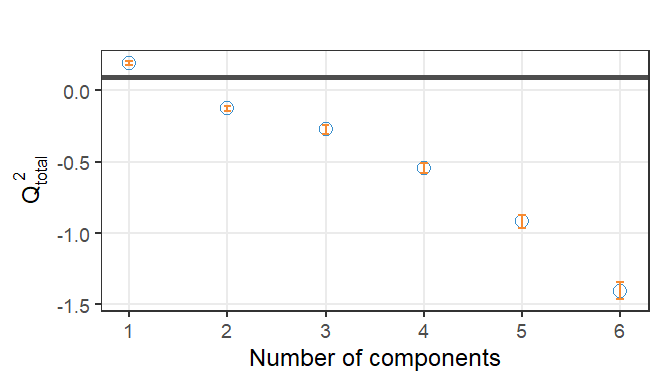
plot(perf.pls.result, criterion = 'Q2.total')

The graph above plots the repeated cross-validation (100 × 10−fold CV) Q2 score for the PLS model. Horizontal line depicts the threshold Q2 = 0.0975, above which the component is considered valid (good at predicting / generalizing one dataset by the other). The red bars represent the variation of Q2 values across the repeated folds

Clustered Image Maps of the gene relative expression and local parameters in the first two PLS components:

C <- cim(pls.result_NA,comp = 1:2, dist.method = c("correlation","correlation"), mapping="XY")

C$mat[1:17,1:6]

|  | imperv | ABPV | SBPV | ER | Apicystis | MIR |
| --- | --- | --- | --- | --- | --- | --- |
| Defensin-1 | 0,37675102 | -0,07565013 | -0,10456675 | -0,3451383 | -0,07677343 | -0,29687531 |
| apidaecin | 0,27539636 | -0,05502062 | -0,07633606 | -0,25231736 | -0,05675961 | -0,21732105 |
| abaecin | 0,26272014 | -0,04793109 | -0,0711853 | -0,24118186 | -0,06464184 | -0,2124343 |
| L-LDH | 0,48104507 | -0,1582753 | -0,1556731 | -0,43420537 | 0,044031636 | -0,30980334 |
| VG | 0,14409411 | -0,04441419 | -0,04555454 | -0,13037812 | 0,006289089 | -0,09616366 |
| hymenoptaecin | 0,2478423 | 0,004856536 | -0,0491653 | -0,23278065 | -0,17630089 | -0,25662351 |
| AADAT | 0,4191932 | -0,23608503 | -0,170921 | -0,36807073 | 0,264435523 | -0,15976057 |
| MRJP | 0,37046 | -0,24134624 | -0,16280066 | -0,32184702 | 0,309018966 | -0,1044659 |
| GOX | 0,21605974 | -0,10081339 | -0,08059857 | -0,19190123 | 0,088232934 | -0,10577408 |
| relish | 0,22806127 | -0,24364778 | -0,13437695 | -0,18815333 | 0,409187593 | 0,042429313 |
| proletess | 0,20607085 | -0,22168974 | -0,12197143 | -0,16984979 | 0,373268157 | 0,04006186 |
| serpin-5 | 0,28945318 | -0,31879981 | -0,17398593 | -0,2377983 | 0,541363929 | 0,064589205 |
| lysozyme-3 | 0,14286984 | -0,13697838 | -0,07855665 | -0,11951296 | 0,220281768 | 0,009002715 |
| apolipoIII | 0,13606235 | -0,13222826 | -0,07545183 | -0,11363187 | 0,213877407 | 0,010568473 |
| PGRPS1 | 0,1893446 | -0,17034343 | -0,10008953 | -0,15956499 | 0,266159914 | -0,0006359 |
| HSP90 | 0,18868687 | -0,27732054 | -0,13838584 | -0,14771764 | 0,512968385 | 0,120138323 |
| PPO | -0,03095678 | -0,25164092 | -0,08404283 | 0,05543021 | 0,600157586 | 0,313900256 |

**Table S13.** PERMANOVA results on the effects of urbanisation level, Apicystis infection, and their interaction on the expression of the AADAT and L-LDH combined with defensin-1 (top), abaecin (middle) or vitellogenin (bottom). P-values were corrected for multiple comparisons using the Benjamini-Hochberg’s method when usefull.

| **Multivariate effects on AADAT, L-LDH and defensin-1 expression** | | | | | | |  |
| --- | --- | --- | --- | --- | --- | --- | --- |
| **Factor** | **Df** | **Sum of sqs** | **R^2^** | **F** | **Pr(>F)** | **P adj** | |
| Urbanisation level | 2 | 54.17 | 0.08385 | 2.6483 | 0.0271 | 0.0813 | |
| Apicystis infection | 1 | 21.35 | 0.03306 | 2.0880 | 0.1200 | 0.1800 | |
| Urb_level:Apicystis | 2 | 28.44 | 0.04403 | 1.3905 | 0.2044 | 0.2044 | |
| Residual | 53 | 542.00 | 0.83906 |  |  |  | |
| Total | 58 | 645.96 | 1.00000 |  |  |  | |

| **Multivariate effects on AADAT, L-LDH, MRJP and abaecin expression** | | | | | |  |
| --- | --- | --- | --- | --- | --- | --- |
| **Factor** | **Df** | **Sum of sqs** | **R^2^** | **F** | **Pr(>F)** | **P adj** |
| Urbanisation level | 2 | 60.90 | 0.06301 | 1.9602 | 0.0950 | - |
| Apicystis infection | 1 | 28.81 | 0.02981 | 1.8548 | 0.1399 | - |
| Urb_level:Apicystis | 2 | 53.54 | 0.05539 | 1.7232 | 0.1639 | - |
| Residual | 53 | 823.34 | 0.85179 |  |  |  |
| Total | 58 | 966.60 | 1.00000 |  |  |  |

| **Multivariate effects on AADAT, L-LDH and vitellogenin expression** | | | | | |  |
| --- | --- | --- | --- | --- | --- | --- |
| **Factor** | **Df** | **Sum of sqs** | **R^2^** | **F** | **Pr(>F)** | **P adj** |
| Urbanisation level | 2 | 329.1 | 0.04719 | 1.3289 | 0.1587 | - |
| Apicystis infection | 1 | 29.1 | 0.00417 | 0.2349 | 0.5394 | - |
| Urb_level:Apicystis | 2 | 52.6 | 0.00754 | 0.2123 | 0.5520 | - |
| Residual | 53 | 6563.4 | 0.94110 |  |  |  |
| Total | 58 | 6974.2 | 1.00000 |  |  |  |

**Table S14.** Model coefficients obtained from GLMMs with Gamma error distribution and log-link function on gene expression of selected genes following PLS analysis and PERMANOVA. Urbanization levels low and medium are tested against urbanization level high.

| **Model** | **Variable** | **Estimate** | **SE** | ***z* value** | **Pr > \| *z* \|** |
| --- | --- | --- | --- | --- | --- |
| *AADAT* |  |  |  |  |  |
|  | Intercept | 1.221 | 0.226 | 5.393 | **< 0.001** |
|  | Apicystis | 0.806 | 0.341 | 2.362 | **0.018** |
|  | ABPV | 0.192 | 0.210 | 0.912 | 0.362 |
|  | SBPV | 0.634 | 0.583 | 1.088 | 0.277 |
|  | Urb level low | −0.935 | 0.263 | −3.554 | **< 0.01** |
|  | Urb level medium | −0.475 | 0.238 | −1.998 | **0.046** |
|  | Api:ABPV | 1.051 | 0.490 | 2.143 | **0.032** |
|  | Api:SBPV | 1.847 | 1.226 | 1.507 | 0.132 |
|  | ABPV:SBPV | 1.358 | 0.884 | 1.536 | 0.125 |
|  | Api:ABPV:SBPV | 2.622 | 1.867 | 1.404 | 0.160 |
| *L-LDH* |  |  |  |  |  |
|  | Intercept | 0.648 | 0.128 | 5.060 | **< 0.001** |
|  | Apicystis | 0.240 | 0.195 | 1.226 | 0.220 |
|  | ABPV | 0.124 | 0.120 | 1.034 | 0.301 |
|  | SBPV | 0.553 | 0.301 | 1.835 | 0.067 |
|  | Urb level low | −0.625 | 0.163 | −3.846 | **< 0.001** |
|  | Urb level medium | −0.328 | 0.153 | −2.142 | **0.032** |
|  | Api:ABPV | 0.335 | 0.290 | 1.155 | 0.248 |
|  | Api:SBPV | 1.102 | 0.640 | 1.722 | 0.085 |
|  | ABPV:SBPV | 0.821 | 0.465 | 1.765 | 0.078 |
|  | Api:ABPV:SBPV | 1.896 | 0.979 | 1.936 | 0.053 |
| *MRJP* |  |  |  |  |  |
|  | Intercept | 0.627 | 0.221 | 2.841 | **0.004** |
|  | Apicystis | 0.443 | 0.320 | 1.384 | 0.167 |
|  | ABPV | −0.104 | 0.207 | −0.502 | 0.615 |
|  | SBPV | 0.233 | 0.512 | 0.455 | 0.649 |
|  | Urb level low | −0.536 | 0.284 | −1.885 | 0.059 |
|  | Urb level medium | −0.187 | 0.267 | −0.702 | 0.483 |
|  | Api:ABPV | 0.576 | 0.486 | 1.185 | 0.236 |
|  | Api:SBPV | 0.379 | 1.075 | 0.352 | 0.725 |
|  | ABPV:SBPV | 0.036 | 0.780 | 0.046 | 0.964 |
|  | Api:ABPV:SBPV | 0.344 | 1.655 | 0.208 | 0.835 |
| *PPO* |  |  |  |  |  |
|  | Intercept | 0.057 | 0.179 | 0.316 | 0.752 |
|  | Apicystis | −0.105 | 0.214 | −0.491 | 0.624 |
|  | ABPV | −0.212 | 0.132 | −1.606 | 0.108 |
|  | SBPV | 0.039 | 0.327 | 0.118 | 0.906 |
|  | Urb level low | 0.101 | 0.243 | 0.416 | 0.678 |
|  | Urb level medium | 0.152 | 0.234 | 0.650 | 0.516 |
|  | Api:ABPV | −0.544 | 0.306 | −1.779 | 0.075 |
|  | Api:SBPV | −0.122 | 0.707 | −0.173 | 0.863 |
|  | ABPV:SBPV | −0.222 | 0.507 | −0.438 | 0.661 |
|  | Api:ABPV:SBPV | −0.302 | 1.039 | −0.291 | 0.771 |
| *Serpin 5* |  |  |  |  |  |
|  | Intercept | 0.489 | 0.144 | 3.399 | **0.001** |
|  | Apicystis | 0.158 | 0.219 | 0.721 | 0.471 |
|  | ABPV | −0.074 | 0.135 | −0.550 | 0.582 |
|  | SBPV | 0.107 | 0.340 | 0.314 | 0.753 |
|  | Urb level low | −0.184 | 0.183 | −1.003 | 0.316 |
|  | Urb level medium | 0.001 | 0.178 | 0.005 | 0.996 |
|  | Api:ABPV | −0.036 | 0.325 | −0.110 | 0.912 |
|  | Api:SBPV | −0.124 | 0.728 | −0.171 | 0.864 |
|  | ABPV:SBPV | −0.282 | 0.524 | −0.538 | 0.590 |
|  | Api:ABPV:SBPV | −0.193 | 1.099 | −0.175 | 0.861 |
| *HSP90* |  |  |  |  |  |
|  | Intercept | 0.616 | 0.182 | 3.380 | **0.001** |
|  | Apicystis | 0.296 | 0.292 | 1.012 | 0.311 |
|  | ABPV | −0.011 | 0.175 | −0.061 | 0.952 |
|  | SBPV | 0.533 | 0.475 | 1.123 | 0.262 |
|  | Urb level low | −0.061 | 0.227 | −0.267 | 0.789 |
|  | Urb level medium | 0.263 | 0.217 | 1.213 | 0.225 |
|  | Api:ABPV | 0.170 | 0.434 | 0.391 | 0.696 |
|  | Api:SBPV | 0.442 | 1.006 | 0.439 | 0.660 |
|  | ABPV:SBPV | 0.004 | 0.711 | 0.006 | 0.995 |
|  | Api:ABPV:SBPV | 0.480 | 1.516 | 0.317 | 0.751 |

Api: abbreviation for *Apicystis* when used in interactions

**Figure S1**

**
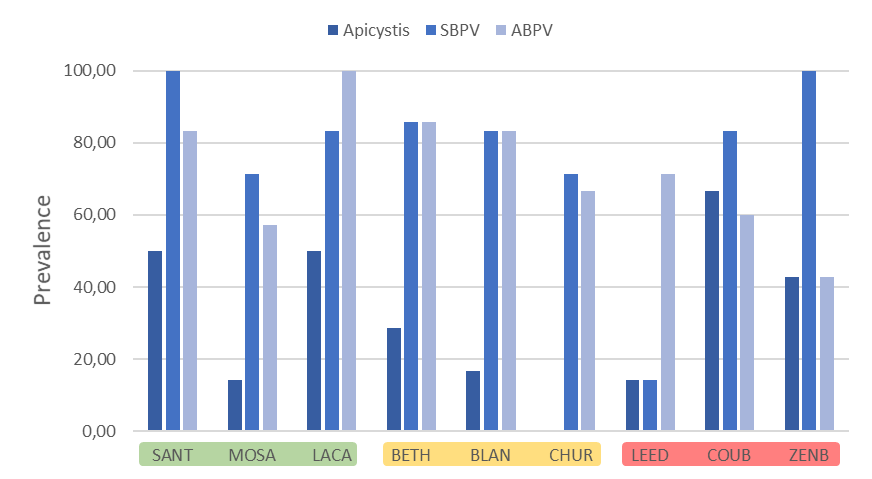
**

Figure S1: Prevalence (% of infected BB) per collection site for *Apicystis bombi*, SBPV, and ABPV; n = 6-8 individuals per site. The colors on the x-axis refer to the local urbanization level (green: low level; yellow: medium level; red: high level).

**
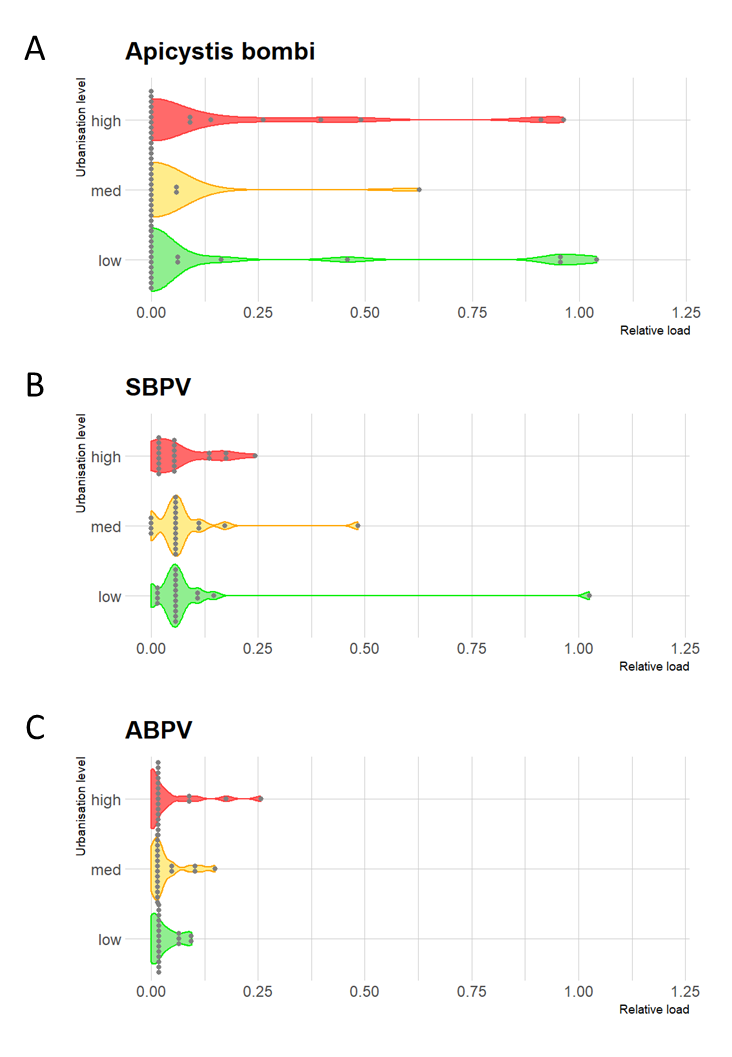
Figure S2**

Figure S2: Relative loads of pathogens detected in BB workers collected along a gradient of urbanization. Individual loads are compared among sampling sites with low, medium (med) or high level of urbanization, for *Apicystis bombi* (A), SBPV (B), and ABPV (C), (low, n = 19; med, n = 20; high, n = 20).

**Figure S3**

**
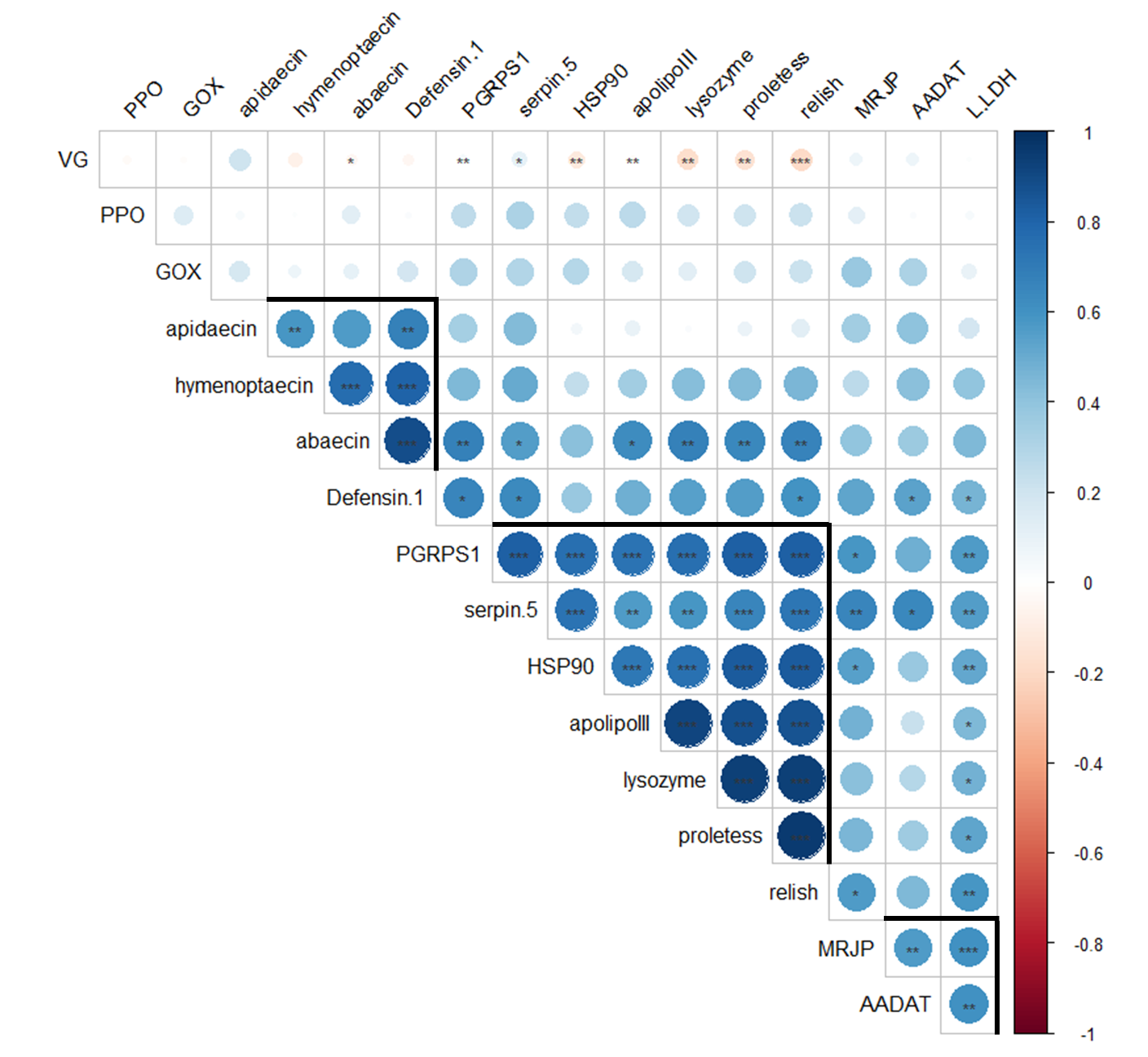
**

Figure S3: Matrix of correlation of the level of expression of the 17 selected immune genes. The areas of circles reflect the absolute value of corresponding Spearman’s correlation coefficients (rho) and colors refer to the heatmap on the right. Asterisks indicate significant correlations (*, P < 0.05; **, P < 0.01; ***, P < 0.001). Bold lines highlight the three groups of genes highly correlated with each other.

**Figure S4**

**
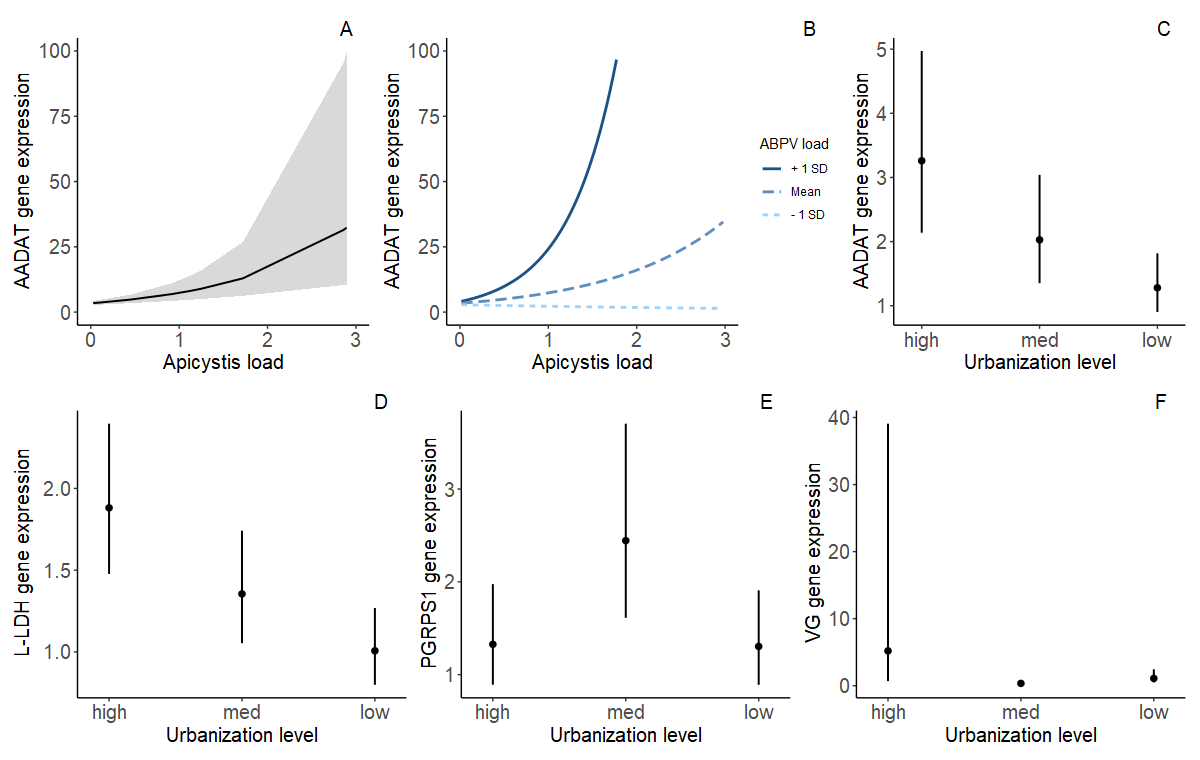
**

Figure S4: Predicted values for correlations between AADAT gene expression and A) Apycistis load, B) Apicystis load mediated by ABPV load (two-way interaction plot), and C) urbanization level; and between D) L-LDH gene expression, E) PGRPS1 gene expression, and F) VG gene expression and urbanization level. Shaded areas and bars represent 95% confidence intervals. Apicystis and ABPV loads were centered and scaled around the mean.

1. Manley, R., Boots, M., Wilfert, L., 2017. Condition-dependent virulence of slow bee paralysis virus in Bombus terrestris: are the impacts of honeybee viruses in wild pollinators underestimated? Oecologia 184, 305–315. https://doi.org/10.1007/s00442-017-3851-2 [↑](#footnote-ref-1)
2. Wilfert, L., Long, G., Leggett, H.C., Schmid-Hempel, P., Butlin, R., Martin, S.J.M., Boots, M., 2016. Deformed wing virus is a recent global epidemic in honeybees driven by *Varroa* mites. Science 351, 594–597. https://doi.org/10.1126/science.aac9976 [↑](#footnote-ref-2)
3. Simmons, W.R., Angelini, D.R., 2017. Chronic exposure to a neonicotinoid increases expression of antimicrobial peptide genes in the bumblebee Bombus impatiens. Sci. Rep. 7, 44773. https://doi.org/10.1038/srep44773 [↑](#footnote-ref-3)
